# Prediction of Sepsis in COVID-19 Using Laboratory Indicators

**DOI:** 10.3389/fcimb.2020.586054

**Published:** 2021-03-02

**Authors:** Guoxing Tang, Ying Luo, Feng Lu, Wei Li, Xiongcheng Liu, Yucen Nan, Yufei Ren, Xiaofei Liao, Song Wu, Hai Jin, Albert Y. Zomaya, Ziyong Sun

**Affiliations:** ^1^ Department of Laboratory Medicine, Tongji Hospital, Tongji Medical College, Huazhong University of Science and Technology, Wuhan, China; ^2^ National Engineering Research Center for Big Data Technology and System, Services Computing Technology and System Lab, Cluster and Grid Computing Lab, School of Computer Science and Technology, Huazhong University of Science and Technology, Wuhan, China; ^3^ The Australia-China Joint Research Centre for Energy Informatics and Demand Response Technologies, Centre for Distributed and High Performance Computing, School of Computer Science, The University of Sydney, Sydney, NSW, Australia; ^4^ Department of Computer Center, Tongji Hospital, Tongji Medical College, Huazhong University of Science and Technology, Wuhan, China

**Keywords:** COVID-19, sepsis, coagulation function, inflammatory factor, artificial intelligence

## Abstract

**Background:**

The outbreak of coronavirus disease 2019 (COVID-19) has become a global public health concern. Many inpatients with COVID-19 have shown clinical symptoms related to sepsis, which will aggravate the deterioration of patients’ condition. We aim to diagnose Viral Sepsis Caused by SARS-CoV-2 by analyzing laboratory test data of patients with COVID-19 and establish an early predictive model for sepsis risk among patients with COVID-19.

**Methods:**

This study retrospectively investigated laboratory test data of 2,453 patients with COVID-19 from electronic health records. Extreme gradient boosting (XGBoost) was employed to build four models with different feature subsets of a total of 69 collected indicators. Meanwhile, the explainable Shapley Additive ePlanation (SHAP) method was adopted to interpret predictive results and to analyze the feature importance of risk factors.

**Findings:**

The model for classifying COVID-19 viral sepsis with seven coagulation function indicators achieved the area under the receiver operating characteristic curve (AUC) 0.9213 (95% CI, 89.94–94.31%), sensitivity 97.17% (95% CI, 94.97–98.46%), and specificity 82.05% (95% CI, 77.24–86.06%). The model for identifying COVID-19 coagulation disorders with eight features provided an average of 3.68 (±) 4.60 days in advance for early warning prediction with 0.9298 AUC (95% CI, 86.91–99.04%), 82.22% sensitivity (95% CI, 67.41–91.49%), and 84.00% specificity (95% CI, 63.08–94.75%).

**Interpretation:**

We found that an abnormality of the coagulation function was related to the occurrence of sepsis and the other routine laboratory test represented by inflammatory factors had a moderate predictive value on coagulopathy, which indicated that early warning of sepsis in COVID-19 patients could be achieved by our established model to improve the patient’s prognosis and to reduce mortality.

## Introduction

The outbreak of coronavirus disease 2019 (COVID-19) in Wuhan, China, has developed into a global pandemic and major public health concern (Tu et al., 2020; Zhou et al., 2020). As of November 23, 2020, around 58 million patients have been diagnosed with severe acute respiratory syndrome coronavirus-2 (SARS-CoV-2) infection, and 14 million (2.37%) patients have died, according to the latest statistical data from Johns Hopkins University. Compared with severe acute respiratory syndrome (SARS) and Middle East Respiratory Syndrome (MERS), SARS-COV-2 infection is less lethal. Due to the high infectivity of this virus, it has however, caused more severe and fatal cases (Tu et al., 2020; Vlachodimitropoulou Koumoutsea et al., 2020). Currently, the cure for COVID-19 is essentially dependent on the patient’s immune system and no specific drugs are available (Cao et al., 2020; The Lancet, 2020). So far, a variety of vaccines have been announced, each with their own good efficacy, but most of them have been released through press releases, and there is still scientific uncertainty (The Lancet, 2020; Nat Nanotechnol, 2020). Therefore, it is crucial to monitor COVID-19 patients closely and to issue an early warning to prevent deterioration.

For COVID-19, in addition to lung injury, impaired liver and kidney function, and microcirculatory dysfunction in some patients fulfilled the criteria synonymous with sepsis and septic shock based on the Sepsis-3 International Consensus (Guan et al., 2020; Li et al., 2020; Zhang et al., 2020). Sepsis is defined as life-threatening organ dysfunction caused by a dysregulated host response to infection (such as bacterial, viral, and/or fungal infections) (Singer et al., 2016). The mortality rate due to sepsis is high, indicating that it is still one of the main causes of death in the world. Identification and treatment of sepsis are a matter of great concern in the medical field and need to be solved urgently (Gaieski et al., 2013; Grondman et al., 2020; Li et al., 2020). A broad range of pathogens can cause sepsis, including bacterial, fungal, or viral pathogens. Although bacterial infections were the main cause of sepsis in these patients, the clinical research and diagnosis of Viral Sepsis still remains very rare (Lin et al., 2018; Musher, 2019; Grondman et al., 2020). Viral Sepsis secondary to viral pneumonia has been reported (Musher, 2019). For patients with COVID-19, secondary Viral Sepsis may be one of the critical causes of patients’ death. The view that the condition of COVID-19 patients is complicated by sepsis, causing aggravation and even death has been widely recognized (Connors and Levy, 2020). In COVID-19, the main reason for this phenomenon is because severe COVID-19 is accompanied by hyper-cytokinemia (Giamarellos-Bourboulis et al., 2020). Tumor necrosis factor-α (TNF-α) and interleukin-6 (IL-6) production by circulating monocytes were persistent, a complex pattern different from influenza or bacterial sepsis (Audo et al., 2020). Furthermore, interleukin-10 (IL-10) has been reported to be a unique feature of the COVID-19 cytokine storm, and its concentrations strongly correlated with those of IL-6 and other inflammatory markers such as C-reactive protein (Lu et al., 2020). The cytokine storm would damage the epithelium of the lungs and lead to extrapulmonary manifestations (cardiovascular, renal, hepatic, gastrointestinal, ocular, dermatologic, and neurological) (Falasca et al., 2020; Johnson et al., 2020; Maxwell et al., 2020). And it induces acute respiratory distress syndrome (ARDS) and secondary sepsis, which often leads to multiorgan failure and death (Lu et al., 2020; Opoka-Winiarska et al., 2020).

With the emerging demands for auxiliary diagnosis and computational tools, several works have been proposed for sepsis prediction in the common medical settings using machine learning. For example, Fohner et al. used latent Dirichlet Allocation as the un-supervised learning model to assess clinical heterogeneity in sepsis, and Taylor et al. applied the random forest model to predict the in‐hospital mortality in emergency department patients with sepsis (Taylor et al., 2016; Fohner et al., 2019). Extreme Gradient Boosting (Xgboost), as it functions as an iterative refit of weak classifiers to residuals of previous models (Yao et al., 2020), has become one of the most popular machine learning models, outperforming other models. It has been widely used in different scenarios in medical application (Li and Zhang, 2020; Ogunleye and Wang, 2020), and there is no exception for sepsis (Zabihi et al., 2019; Yao et al., 2020). To our knowledge, there is no analytical tool to predict which COVID-19 patients are most likely to develop sepsis in the near future. Furthermore, explainable machine learning is the future direction in the medical application as it can offer more credible and traceable outcomes for clinicians (Tonekaboni et al., 2019). As a model which explains unrelated methods, SHAP (Lundberg and Lee, 2017) started to draw the attention of researchers gradually.

To our knowledge, there is no analytical tool that predicts which COVID-19 patients are most likely to develop sepsis in the near future. Our research aims to use interpretable machine learning to identify risk factors for Viral Sepsis Caused by SARS-CoV-2 (VSCS-2) and to rationalize these indicators using knowledge about viral sepsis. On this basis, the laboratory indicators used for early warning of VSCS-2 were developed and provided some enlightenment for the study of respiratory viruses. Predictive models were established to predict coagualopathy using the laboratory indicators, and issue warning for the early diagnosis and treatment of VSCS-2 to allow better prognosis.

## Methods

### Materials

This study was carried out at Tongji Hospital (the largest hospital in central China) and was approved by the ethical committee of Tongji Hospital, Tongji Medical College, Huazhong University of Science and Technology, China. A total of 2,453 patients with COVID-19 (1,257 males, 1,196 females) were recruited between December 2019 and March 2020. These patients were diagnosed with nucleic acid testing or clinical diagnosis. The age distribution of the 2,453 patients with COVID-19 is 55.7 ± 15.3 years old.

### Identification of Risk Factors for VSCS-2

We used Extreme gradient boosting (Xgboost) (Chen and Guestrin, 2016), which has been actively promoted in the medical community, and the Shapley Additive ePlanation (SHAP) (Lundberg and Lee, 2017) method, a tool for analyzing the impact of each feature on the prediction, to find the most relevant risk factors for VSCS-2.

**Figure 1:**
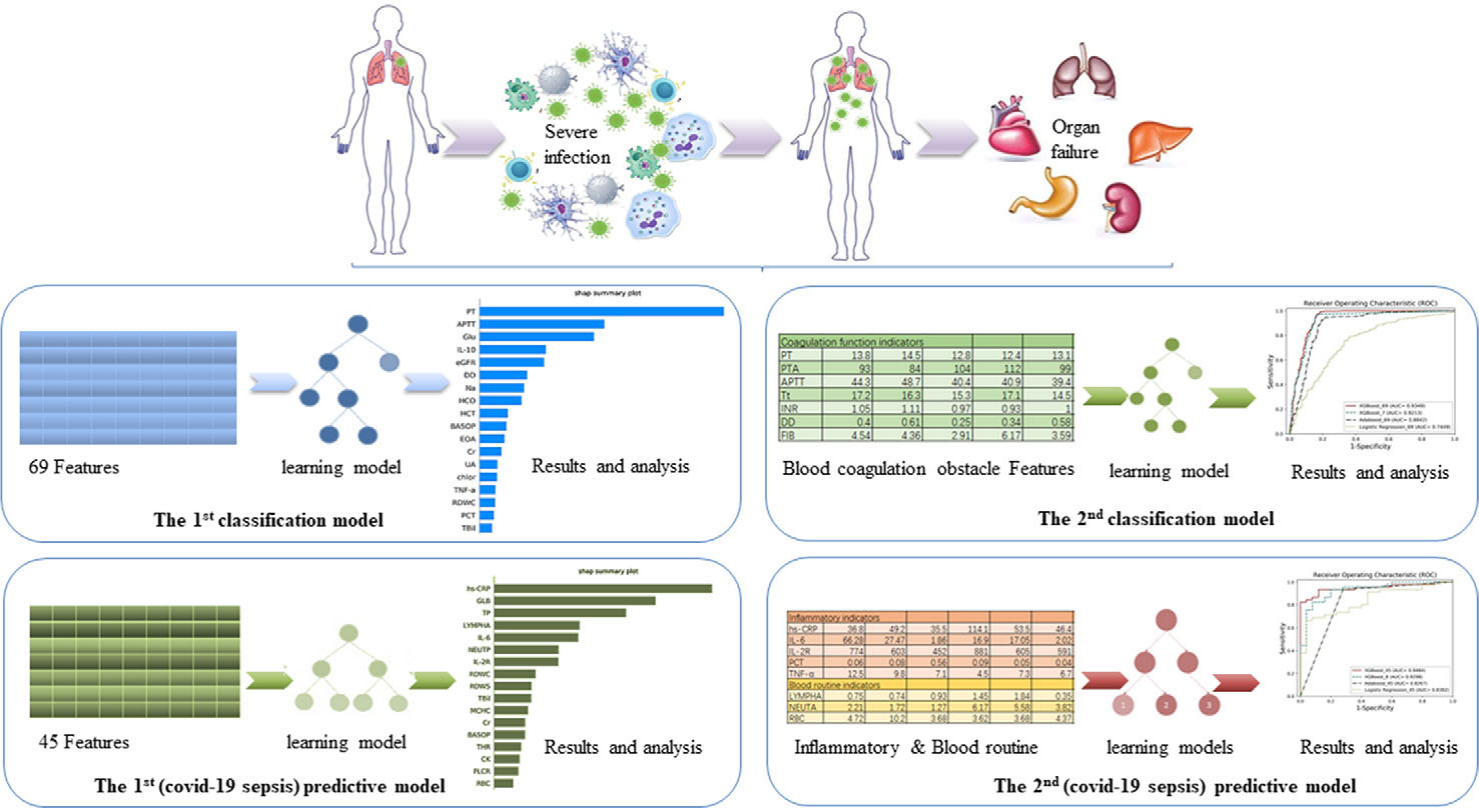
The overview of the four models. The aim of the first classification model for the early identification of VSCS-2 is to identify the risk factors. The second classification model for VSCS-2 is to further clarify the relationship between seven coagulation indicators and VSCS-2. The first predictive model is to classify and predict COVID-19 coagulopathy, which hints VSCS-2 from coagulation disorder. The second predictive model implemented the prediction of coagulation disorder with as few inflammatory and blood routine indicators as possible.

We built the first classification model to have a general understating of VSCS-2 (**Figure 1**). According to sepsis-1 criteria (**Table 1**), the recruited COVID-19 patients were classified into two groups: VSCS-2 group (1,376 patients, 56.1%) and pure COVID-19 patient group (1,077 patients, 43.9%). Due to the complex pathogens of sepsis, this study tries to incorporate selected laboratory test items with clinical signs and symptoms. They are inflammatory factors, coagulation factors, and blood routines that may favor the occurrence of VSCS-2. Additionally, the biochemical blood indicators that can indicate the function of the pancreas, liver, kidney, glucose metabolism, and myocardial injury are also included. The total 69 indicators, are classified into four types (**Supplementary Table 1**). All the indicators of patients were extracted from their electronic health records. Here, 2,453 samples were divided according to 7:3, with 1,717 cases in the training set and 736 cases in the testing set.

**Table 1:** Systemic Inflammatory Response Syndrome (SIRS) criteria.

Meeting at least two of the four criteria
(1) body temperature > 38.3 or <36.3°C	
(2) heart rate > 90 beats/min	
(3) respiration rate > 20 breaths/min or PaCO_2_ < 32mmHg
(4) white cell count > 12xl0^9^ cells/L or < 4xl0^9^ cells/L	

Based on the knowledge of viral sepsis and the results of the first classification model, we noted a strong correlation between coagulation disorders and VSCS-2. To verify our hypothesis, seven coagulation function indicators were used to build the second classification model to explore the link between coagulation function and VSCS-2. The indicators are prothrombin time (PT), prothrombin activity (PTA), activated partial thromboplastin time (APTT), thrombin time (Tt), international standardized ratio (INR), D-dimer (DD), and fibrinogen (FIB). The population was also divided into the training set and the testing set according to the 7:3 ratio.

### Predictive Models for Coagulation Disorder

To further analyze the factors related to coagulation disorders, we used the coagulation function factors to re-evaluate the 2,453 patients with COVID-19 and to identify whether they had coagulation disorders. If one of the following criteria (PT > 14.5 seconds; DD > 0.5 ug/mL; APTT > 42 seconds; and PTA < 75%) is met, the patient was considered as having coagulation disorder. Otherwise, the patient is considered to have normal blood coagulation. Finally, 988 patients with COVID-19 were labeled as having abnormal coagulation function, while 510 patients had normal coagulation function, giving a total of 1,498 patients. From the 69 indicators (see **Supplementary Table 1**), 22 blood routine factors, eight inflammatory factors, and 15 selected blood biochemistry indicators, a total of 45 features were used to build the first predictive model to classify and predict COVID-19 coagulopathy. This model aimed to find laboratory indicators that have an important impact on COVID-19 coagulopathy.

To identify the predictive ability of the laboratory test factors for coagulation dysfunction, we randomly extracted 70 samples from 1,498 patients whose detection time of inflammatory and blood routine factors was before the detection time of coagulation. This time interval between the inflammatory and coagulation factors forms an observation window of the disease course. The 70 samples were used as the testing set, of which 45 are abnormal blood coagulation samples, and 25 are normal coagulation samples. Finally, the training set contains 1,428 samples, including 943 abnormal coagulation samples and 485 normal coagulation samples.

To determine the critical risk factors of coagulation dysfunction with clinical significance, we selected the most important features based on the first prediction model and the analysis results of the SHAP method. To verify the effect of these features and to provide a clinical reference, we developed the second predictive model for COVID-19 coagulopathy.

## Results

**Figure 2:**
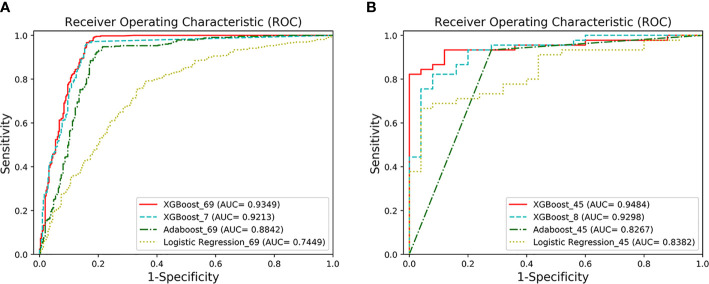
The ROC curves of the four models. **(A)** AUCs of the two classification models under the ROC curve. XGBoost_69 refers to the ROC curve of the first classification model, and XGBoost_7 refers to the ROC curve of the second classification model. The performance of XGFBoost_7 model (long dotted line) is very close to the performance of XGBoost_69 model (solid line). The performance of the XGBoost models is superior to Adaboost_69 and Logistic Regression_69. **(B)** AUCs of the two prediction models under the ROC curve. XGBoost_45 represents the ROC curve of the first predictive model, and XGBoost_8 represents the ROC curve of the second predictive model. The performance of XGBoost_8 model (long dotted line) is very close to XGBoost-45 (solid line).

The detailed demographics and laboratory characteristics distribution are shown below in **Table 2**. The classification and predictive performance of the four models are shown in **Figure 2** and **Table 3**.

**Table 2:** Demographic and laboratory characteristics of the included participants.

Characteristics	Overall
Age, mean±std years	55.7±15.3
Gender, n (%)	
Male	1257 (51.2)
Female	1196 (48.8)
Laboratory test, means±sd,	
Hs-CRP (mg/L)	32.28±47.81
PCT (ng/mL)	0.23±1.59
IL-6 (pg/ml)	28.97±220.67
IL-1 (pg/ml)	6.45±7.84
TNF-α (pg/ml)	8.73±4.49
IL-2R (U/mL)	599.45±411.67
IL-8 (pg/ml)	19.25±38.75
IL-10 (pg/ml)	6.50±5.93
PT (s)	13.87±1.44
PTA (%)	92.40±13.10
APTT (s)	39.60±6.96
Tt (s)	16.82±4.19
INR	1.07±0.15
DD (μg/mL)	1.86±4.07
FIB (g/L)	4.47±1.46
RBC(*10^12/L)	4.67±2.9
WBC (*10^9/L)	6.59±6.73
NEUTA (*10^9/L)	4.06±2.37
NEUTP (%)	65.00±12.74
LYMPHA (*10^9/L)	1.34±0.61
PLT (*10^9/L)	227.75±89.83
GLB (g/L)	31.87±5.35
ALT (U/L)	30.51±33.24
PAB (mg/L)	209.31±87.52
TBil (μmol/L)	10.21±13.77
TP (g/L)	69.15±6.12
AST(GOT) (U/L)	30.32±25.12
Urea (mmol/L)	278.31±100.28
UA (μmol/L)	5.00±3.24
Egfr (ml/min/1.73m^2)	90.98±21.18
CK (U/L)	101.95±177.31

**Table 3:** Performance of the four models.

	Accuracy (%)	AUC	95% CI for AUC (%)	Sensitivity (%)	95% CI for sensitivity (%)	Specificity (%)	95% CI for specificity (%)	F1(%)
The 1^st^ classification model	91.03	0.9349	91.42–95.56	96.93	94.68– 98.29	83.01	78.28 –86.92	92.57
The 2^nd^ classification model	90.76	0.9213	89.94–94.31	97.17	94.97– 98.46	82.05	77.24– 86.06	92.38
The 1^st^ predictive model	88.57	0.9484	89.71–99.97	88.89	75.15– 95.83	88.00	67.64– 98.85	90.91
The 2^nd^ predictive model	82.86	0.9298	86.91–99.04	82.22	67.41– 91.49	84.00	63.08– 94.75	86.05

### Correlation Between Coagulopathy and VSCS-2

**Figure 3:**
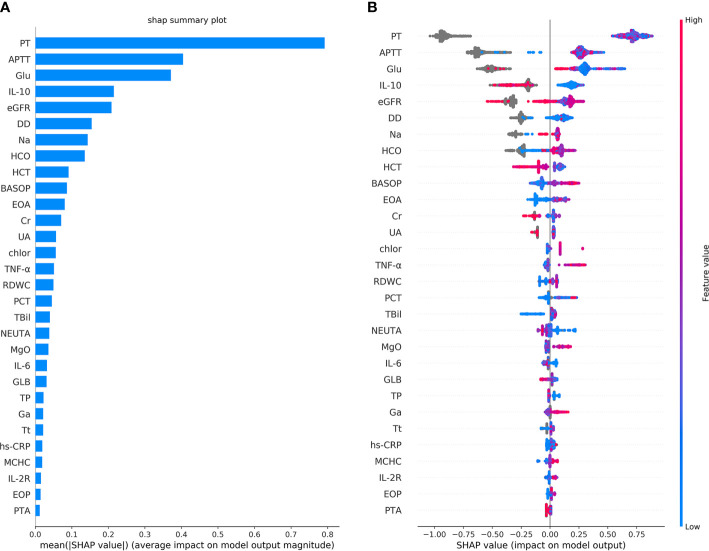
The top 30 features importance were obtained by the SHAP analysis in the first classification model. Here, a total of 69 features were used to classify 1,376 cases of COVID-19 sepsis and 1,077 cases of COVID-19 non-sepsis. **(A)** The features importance of the first classification model based on additivity (shows the top 30 out of 69). The Y-axis is the feature. The abscissa shows the importance of each feature, given by the mean value (SHAP value) of the absolute value of a feature’s influence on the target variable. It suggests the influence of features on model classification. **(B)** The overall analysis of the influence of each feature (also shows the top 30 only). It indicates not only the influence of features but also represents how the influence is impacted. Each row in the figure represents a feature, and the abscissa is the SHAP value. A point represents a sample. The color closed to red indicates the larger value, while the color closed to blue indicates the smaller value. For example, IL-10 is an essential feature and is negatively correlated with VSCS-2, which is, the smaller the value, the higher chance for the determination of VSCS-2.

The classification performance of the first classification model (**Table 3**) shows that the AUC of the model under ROC curve was 0.9349 (95% CI, 91.42–95.56%), and the sensitivity and specificity were 96.93% (95% CI, 94.68–98.29%) and 83.01% (95% CI, 78.28–86.92%), respectively. Then, the critical risk factors of the model are explained by the SHAP method. The results (**Figure 3**) suggest that there is a strong correlation between the coagulation function indicators and VSCS-2. The largest contribution to the model was PT, the second was APTT, and the sixth was DD. The AUC performance, 0.9213 (95% CI, 89.94–94.31%), of the second classification model (**Table 3**) also hinted to the correlation between coagulation function indicators and VSCS-2. As shown in **Figure 2**, the classification performance of VSCS-2 using seven coagulation indicators is very close to the model using all 69 indicators. The result also shows that a variety of biochemistry and blood routine indicators have a strong correlation with VSCS-2, such as estimated Glomerular filtration rate (eGFR), Hematocrit (HCT), Creatinine (Cr), and Total bilirubin (TBil).

The results of the single-factor analysis using the SHAP method show (**Supplementary Figure 1**) that the impaired coagulation function indicated VSCS-2. That is, the value of PT is roughly higher than 12s, the APTT is above about 35s, and the value of DD is almost higher than 0.5mg/L. These values are almost consistent with the clinical detection of coagulation dysfunction. **Figure 3** also shows that there is a strong correlation between inflammatory indicators and VSCS-2, for example IL-10, TNF-α, IL-6, hypersensitive C-reactive protein (hs-CRP), and Interleukin 2 Receptor (IL-2R). From the relevant analysis of the single-factor analysis (**Supplementary Figure 1**), it is suggested that the performance of PT is closely related to IL-6, TNF-α, and Interleukin 8 (IL-8). The performance of APTT is closely related to Glucose (Glu), calibration Calcium (cCa), and estimated Glomerular filtration rate (eGFR). The DD is closely related to Basophil percentage (BASOP), Eosinophilia percentage (EOP), and Eosinophilia absolute value (EOA).

### Inflammatory Indicators Predict Coagulation Disorder

**Figure 4:**
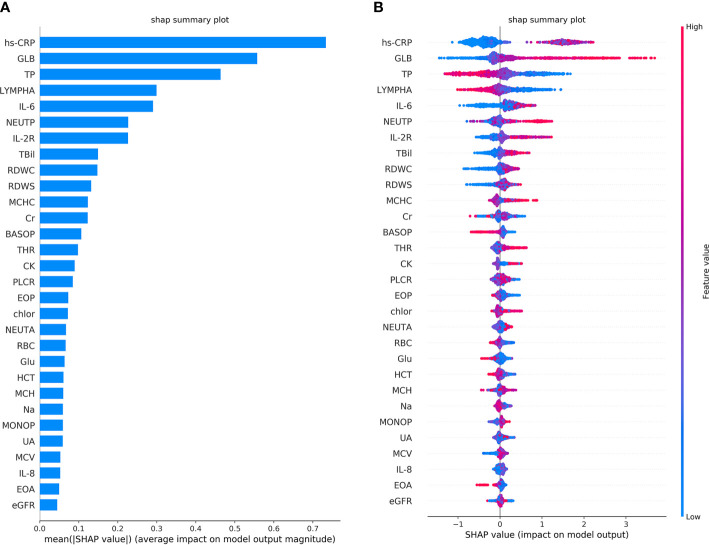
The top 30 features importance were obtained by SHAP analysis in the first predictive model. A total of 45 features were used to classify 953 cases of COVID-19 coagulation dysfunction and 1,077 cases of normal COVID-19 coagulation. **(A)** The features importance of the first predictive model based on additivity (take the top 30). **(B)** The overall analysis of the influence of each feature (take the top 30).

**Figure 5:**
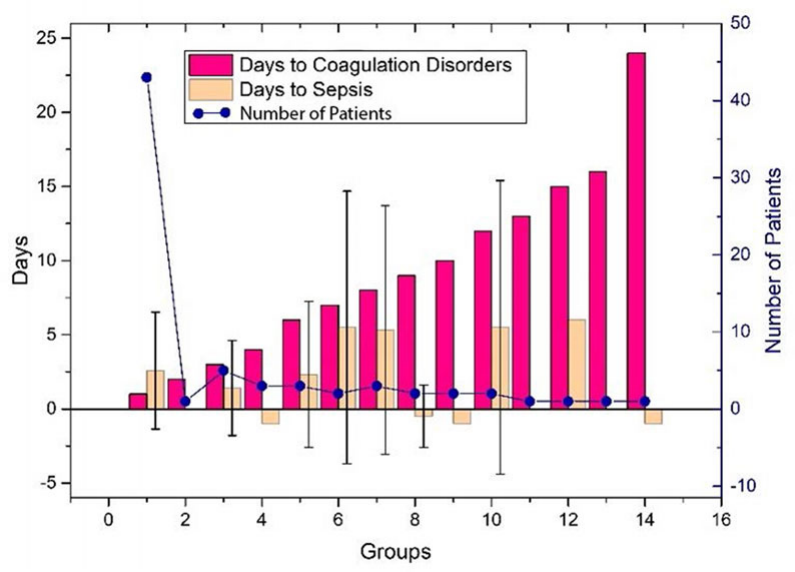
The days of the prediction between the detection of inflammatory factors and the time occurring coagulation disorders, or VSCS-2 for the 70 patients. The red bar shows the time difference between the detection of inflammatory factors and coagulation disorders, and the positive value indicates that it can be predicted in advance. The yellow bar gives the time difference between the detection of inflammatory factors and VSCS-2. Here, we use sepsis-1 criteria (**Table 1**) to identify VSCS-2. Negative values indicate that VSCS-2 occurs before. The blue dots represent the number of patients confirmed on that day. As shown in the figure, the number of people who predicted one day in advance was the highest, 43, while the remaining cases did not exceed five days. Here, the detection time of laboratory indicators (including inflammatory factors) can only be obtained retrospectively at the time point with complete test data. During the COVID-19 outbreak, the tests were usually cases dependent, and some data is missing.

The performance of the first predictive model (**Table 3**) shows that the AUC, sensitivity, and specificity reaches 0.9484 (95% CI, 89.71–99.97%), 88.89% (95% CI, 75.15–95.83%), and 88.00% (95% CI, 67.64–98.85%), respectively. The SHAP analysis (**Figure 4**) of the model suggested that the inflammatory indicators, hs-CRP, IL-6, and IL-2R, are significantly suggestive of coagulation disorders. Furthermore, Immune-related blood routine indicators globulin (GLB), Total protein (TP), Lymphocyte absolute value (LYMPHA), and Neutrophil percentage (NEUTP) also rank at the forefront. According to the statistical analysis of 70 test samples, the model can provide an average of 3.68 (±) 4.60 days (**Figure 5**) in advance early warning of COVID-19 coagulation disorders.

From the single-factor analysis results (**Supplementary Figure 2**), the trend boundary of hs-CRP is obvious. The value of 40±5 mg/L or even more increases the probability of coagulation disorder, and if it is below this threshold, the risk of abnormal coagulation is relatively low. If IL-6 is above 10–15 pg/mL, or IL-2R above 600 μ/mL, the probability of coagulopathy risk is higher. If IL-6 is below 10–15 pg/mL, or IL-2R below 500 μ/mL, the probability is lower. Moreover, all of the LYMPHA below 1.5 10^9/L, GLB above 28 g/L, or TP below 70 g/L can positively indicate the risk of VSCS-2.

In the second prediction model, we used the ablation experiment method to successfully add the crucial features obtained from **Figure 4** and the medical analysis. The XGBoost is used to analyze the performance of the models (**Table 4**). The SHAP method is applied to analyze the influence of eight features on the prediction results (**Supplementary Figure 3**) and the single-factor influence of the features (**Supplementary Figure 4**).

**Table 4:** The ablation experiments of the second predictive model.

	Accuracy (%)	AUC (%)	95% CI for AUC (%)	Sensitivity (%)	95% CI for sensitivity (%)	Specificity (%)	95% CI for specificity (%)	F1 (%)
hs-CRP	68.75	81.28	71.2–91.27	66.67	50.95–79.56	72.00	50.40–87.13	73.17
+IL-6	74.29	79.56	68.92–90.19	80.00	64.95–89.91	64.00	42.62–81.29	80.00
+IL-2R	75.71	83.38	74.00–92.76	77.78	62.52–88.29	72.00	50.41–87.12	80.46
+PCT	75.71	79.56	68.96–90.15	75.56	60.14–86.61	76.00	54.48–89.84	80.00
+TNF-α	74.29	83.91	74.60–93.22	71.11	55.48–83.16	80.00	58.87–92.39	78.05
+LYMPHA	78.57	87.38	78.88–95.88	73.33	57.79–84.90	88.00	67.66–96.85	81.48
+NEUTP	80.00	90.58	83.59–97.56	73.33	57.79–84.90	92.00	72.50–98.60	82.50
+RBC	82.86	92.98	86.91–99.04	82.22	67.41–91.49	84.00	63.08–94.75	86.05

The first experiment only used the hs-CRP value as a single input feature of the model. Then IL-6 was added to the second column, that is, the model used hs-CRP and IL-6 as input features. Finally, when taking eight features, including Red Blood Cell Count (RBC), the model’s performance is very close to the prediction model with 45 features.

## Discussion

Sepsis is a concerning public health problem as the host’s response to the source of infection results in significant morbidity and mortality (Kempker et al., 2018; Alhazzani et al., 2020). Currently, sepsis with subsequent multiorgan dysfunction is one of the main causes of death in COVID-19 patients, and several previous studies have looked at the question of activation of the coagulation system in advanced or severe patients with SARS-CoV-2 infection (Li et al., 2020; Tang et al., 2020). Reliable definitions and increased attention are of utmost necessity in the medical domain, as proper and early treatment of illness demands an accurate preceding diagnosis (Fan et al., 2016). Therefore, novel technologies and detection methods allow for the rapid and accurate identification of sepsis, or even coagulopathy in patients with SARS-CoV-2 infection are especially urgently needed for the control and management of the disease in clinical practice (Channappanavar et al., 2016).

The present study enrolled approximately 2,500 patients to examine the feasibility and efficiency of measuring markers in routine laboratory tests for the diagnosis and prediction of sepsis in patients with SARS-CoV-2 infection. It was found that the abnormality of the coagulation function highly suggested the occurrence of sepsis and the other parameters represented by inflammatory factors including IL-2R, IL-6, and hs-CRP had a moderate predictive value on coagulopathy. The established model using the combination of former markers such as inflammatory indexes had the potential for the early warning of sepsis in COVID-19 patients. It could help and guide clinicians to conduct available measures to improve the patient’s prognosis. To the best of our knowledge, this study is the first clinical evaluation targeted at the early diagnosis of sepsis in patients with COVID-19 using machine learning.

The existing shreds of evidence do not answer the question of which patients overreact in terms of hyper-inflammation and “cytokine storm”; although other people have slight signs, the same microorganism may be found on their airways. So, it is exciting and meaningful to contemplate and speculate on the reasons for the various patterns of change before the occurrence of sepsis in SARS-CoV-2 infection.

It has been well studied that cytokines play an essential part in the immune system during viral infections. A fast and effectively organized intuitive immune response is the vanguard of defense against viral infections. But an imbalanced and over immune response can lead to damage to the immune organism (Law et al., 2005; Tynell et al., 2016; Ye et al., 2020). *In vitro* experiments have shown that after SARS infection, mainly airway epithelial cells, dendritic cells, monocytes, and macrophages participate in the release of chemokines and cytokines (Scheuplein et al., 2015; Tynell et al., 2016). After MERS infection, plasma cell-like dendritic cells are mainly involved in the release of chemokines and cytokines (Giannakopoulos et al., 2017). According to our data, after SARS-CoV-2 infection, the generation of inflammatory storms is mainly involved in T lymphocytes. Sepsis might be associated with endogenous activation of coagulation and fibrinolysis during COVID-19. Several studies demonstrated that dysregulation of procoagulant and fibrinolytic pathways may uniquely contribute to the pathophysiology of sepsis. However, this issue should be further investigated to obtain more details (Bouck et al., 2020; Colantuoni et al., 2020; Jose et al., 2020; Kang et al., 2020).

Based on our findings, we think that the former activated inflammation may be the forerunner of later coagulopathy, which further degenerates into septic shock and finally causes multi organ dysfunctions. This assumption is consistent with the previous theory that reducing inflammation as one of the conventional methods to sepsis pathophysiology and resilience is considered an intuitive way in which organisms respond to microorganisms (Gotts and Matthay, 2016; Rosen et al., 2019). The intrinsic mechanism might be that the cell subsets mainly composed of lymphocytes are activated and secrete cytokines and chemokines during the body’s early immune response, then gradually become exhausted and tent to apoptosis (Mira et al., 2017; Patel et al., 2019). Multiple organs of the patient are damaged due to excessive inflammation and disorder of the endogenous coagulation activation pathway (Gomez and Kellum, 2016; Shen et al., 2019). All of the above finally leads to the collapse of the body’s homeostasis.

There are also some limitations in this study. First, the scale of patients included in this study is reasonably large, but they all come from a single center. Further validation in more centers with more patient cases and complete laboratory test data need to be verified in the future. Second, even though the current study adds to the understanding of the progress of sepsis syndromes, this is a retrospective study and lacks verification *in vivo*. There is much work yet to be performed to understand these changes entirely. Finally, the lack of some test items may cause a bias and subsequently misleading results, so the conclusions we obtained in this study need to be confirmed in a prospective design.

In summary, our study provides a preliminary understanding of sepsis in patients with SARS-CoV-2 infection. We found that inflammation and coagulopathy might play a prominent part as a precursor in progress. We envisage that our findings could serve as an instrumental tool for diagnosing and predicting sepsis during COVID-19 treatment.

## Data Availability Statement

The original contributions presented in the study are included in the article/**Supplementary Material**. Further inquiries can be directed to the corresponding author.

## Ethics Statement

The studies involving human participants were reviewed and approved by the ethical committee of Tongji Hospital, Tongji Medical College, Huazhong University of Science and Technology, China. The patients/participants provided their written informed consent to participate in this study.

## Author Contributions

ZS, HJ, and AZ were responsible for leading this study. GT, YL, FL, and WL were responsible for discussing and designing the research plan. ZS, GT, and YL were responsible for providing the clinical dataset and interpretations of the laboratory analysis results. XioL, YN, YR, XiaL, and SW analyzed and designed the machine learning models. FL, WL, and XioL were responsible for preprocessing the dataset, developing and implementing algorithm details, and conducting the analysis on the dataset. GT, YL, FL, WL, and XioL were responsible for writing the manuscript and guarantee the data, analysis, and interpretation. All authors contributed to the article and approved the submitted version.

## Funding

Hubei Provincial Development and Reform Commission Program “Hubei Big Data Analysis Platform and Intelligent Service Project for Medical and Health” and the National Mega Project on Major Infectious Disease Prevention (2017ZX10103005-007).

## Conflict of Interest

The authors declare that the research was conducted in the absence of any commercial or financial relationships that could be construed as a potential conflict of interest.

## References

[B1] Alhazzani W. Moller M. H. Arabi Y. M. Loeb M. Gong M. N. Fan E.. (2020). Surviving Sepsis Campaign: Guidelines on the Management of Critically Ill Adults with Coronavirus Disease 2019 (COVID-19). Crit. Care Med. 48, e440–e469. 10.1097/CCM.0000000000004363 32224769 PMC7176264

[B2] Audo A. Bonato V. Cavozza C. Maj G. Pistis G. Secco G. G. (2020). Acute Pulmonary Embolism in SARS-CoV-2 Infection Treated with Surgical Embolectomy. Ann. Thorac. Surg. 110, e403–e404. 10.1016/j.athoracsur.2020.04.013 32360384 PMC7187836

[B3] Bouck E. G. Denorme F. Holle L. A. Middelton E. A. Blair A. De Laat B.. (2020). COVID-19 and Sepsis Are Associated With Different Abnormalities in Plasma Procoagulant and Fibrinolytic Activity. Arterioscler. Thromb. Vasc. Biol. 41, 401–414. 10.1161/ATVBAHA.120.315338 33196292 PMC7942774

[B4] Cao Y. Wu H. Zhai W. Wang Y. Li M. Li M.. (2020). A safety consideration of mesenchymal stem cell therapy on COVID-19. Stem Cell Res. 49, 102066. 10.1016/j.scr.2020.102066 33242791 PMC7585498

[B5] Channappanavar R. Fehr A. R. Vijay R. Mack M. Zhao J. Meyerholz D. K.. (2016). Dysregulated Type I Interferon and Inflammatory Monocyte-Macrophage Responses Cause Lethal Pneumonia in SARS-CoV-Infected Mice. Cell Host. Microbe 19, 181–193. 10.1016/j.chom.2016.01.007 26867177 PMC4752723

[B6] Chen T. Guestrin C. (2016). “XGBoost,” in Proceedings of the 22nd ACM SIGKDD International Conference on Knowledge Discovery and Data Mining (New York, NY, United States: Association for Computing Machinery). 10.1145/2939672.2939785

[B7] Colantuoni A. Martini R. Caprari P. Ballestri M. Capecchi P. L. Gnasso A.. (2020). COVID-19 Sepsis and Microcirculation Dysfunction. Front. Physiol. 11, 747. 10.3389/fphys.2020.00747 32676039 PMC7333313

[B8] Connors J. M. Levy J. H. (2020). COVID-19 and its implications for thrombosis and anticoagulation. Blood 135, 2033–2040. 10.1182/blood.2020006000 32339221 PMC7273827

[B9] Falasca L. Nardacci R. Colombo D. Lalle E. Di Caro A. Nicastri E.. (2020). Postmortem Findings in Italian Patients With COVID-19: A Descriptive Full Autopsy Study of Cases With and Without Comorbidities. J. Infect. Dis. 222, 1807–1815. 10.1093/infdis/jiaa578 32914853 PMC7543426

[B10] Fan S. L. Miller N. S. Lee J. Remick D. G. (2016). Diagnosing sepsis - The role of laboratory medicine. Clin. Chim. Acta 460, 203–210. 10.1016/j.cca.2016.07.002 27387712 PMC4980259

[B11] Fohner A. E. Greene J. D. Lawson B. L. Chen J. H. Kipnis P. Escobar G. J.. (2019). Assessing clinical heterogeneity in sepsis through treatment patterns and machine learning. J. Am. Med. Inf. Assoc. 26 (12), 1466–1477. 10.1093/jamia/ocz106 PMC7647146 31314892

[B12] Gaieski D. F. Edwards J. M. Kallan M. J. Carr B. G. (2013). Benchmarking the incidence and mortality of severe sepsis in the United States. Crit. Care Med. 41, 1167–1174. 10.1097/CCM.0b013e31827c09f8 23442987

[B13] Giamarellos-Bourboulis E. J. Netea M. G. Rovina N. Akinosoglou K. Antoniadou A. Antonakos N.. (2020). Complex Immune Dysregulation in COVID-19 Patients with Severe Respiratory Failure. Cell Host. Microbe 27, 992–1000. 10.1016/j.chom.2020.04.009 32320677 PMC7172841

[B14] Giannakopoulos K. Hoffmann U. Ansari U. Bertsch T. Borggrefe M. Akin I.. (2017). The Use of Biomarkers in Sepsis: A Systematic Review. Curr. Pharm. Biotechnol. 18, 499–507. 10.2174/1389201018666170601080111 28571560

[B15] Gomez H. Kellum J. A. (2016). Sepsis-induced acute kidney injury. Curr. Opin. Crit. Care 22, 546–553. 10.1097/MCC.0000000000000356 27661757 PMC5654474

[B16] Gotts J. E. Matthay M. A. (2016). Sepsis: pathophysiology and clinical management. BMJ 353, i1585. 10.1136/bmj.i1585 27217054

[B17] Grondman I. Pirvu A. Riza A. Ioana M. Netea M. G. (2020). Biomarkers of inflammation and the etiology of sepsis. Biochem. Soc. Trans. 48, 1–14. 10.1042/BST20190029 32049312

[B18] Guan W. J. Ni Z. Y. Hu Y. Liang W. H. Ou C. Q. He J. X.. (2020). Clinical Characteristics of Coronavirus Disease 2019 in China. N. Engl. J. Med. 382, 1708–1720. 10.1056/NEJMoa2002032 32109013 PMC7092819

[B19] Johnson K. D. Harris C. Cain J. K. Hummer C. Goyal H. Perisetti A. (2020). Pulmonary and Extra-Pulmonary Clinical Manifestations of COVID-19. Front. Med. (Lausanne) 7, 526. 10.3389/fmed.2020.00526 32903492 PMC7438449

[B20] Jose R. J. Williams A. Manuel A. Brown J. S. Chambers R. C. (2020). Targeting coagulation activation in severe COVID-19 pneumonia: lessons from bacterial pneumonia and sepsis. Eur. Respir. Rev. 29, 200240. 10.1183/16000617.0240-2020 33004529 PMC7537941

[B21] Kang S. Tanaka T. Inoue H. Ono C. Hashimoto S. Kioi Y.. (2020). IL-6 trans-signaling induces plasminogen activator inhibitor-1 from vascular endothelial cells in cytokine release syndrome. Proc. Natl. Acad. Sci. U. S. A. 117, 22351–22356. 10.1073/pnas.2010229117 32826331 PMC7486751

[B22] Kempker J. A. Wang H. E. Martin G. S. (2018). Sepsis is a preventable public health problem. Crit. Care 22, 116. 10.1186/s13054-018-2048-3 29729670 PMC5936625

[B23] Law H. K. Cheung C. Y. Ng H. Y. Sia S. F. Chan Y. O. Luk W.. (2005). Chemokine up-regulation in SARS-coronavirus-infected, monocyte-derived. Blood 106, 2366–2374. 10.1182/blood-2004-10-4166 15860669 PMC1895271

[B24] Li S. Zhang X. (2020). Research on orthopedic auxiliary classification and prediction model based on XGBoost algorithm. Neural Comput. Applic. 32, 1971–1979. 10.1007/s00521-019-04378-4

[B25] Li H. Liu L. Zhang D. Xu J. Dai H. Tang N.. (2020). SARS-CoV-2 and viral sepsis: observations and hypotheses. Lancet 395, 1517–1520. 10.1016/S0140-6736(20)30920-X 32311318 PMC7164875

[B26] Lin G. L. Mcginley J. P. Drysdale S. B. Pollard A. J. (2018). Epidemiology and Immune Pathogenesis of Viral Sepsis. Front. Immunol. 9, 2147. 10.3389/fimmu.2018.02147 30319615 PMC6170629

[B27] Lu L. Zhang H. Dauphars D. J. He Y. W. (2020). A Potential Role of Interleukin 10 in COVID-19 Pathogenesis. Trends Immunol. 42, 3–5. 10.1016/j.it.2020.10.012 33214057 PMC7605819

[B28] Lundberg S. M. Lee S.-I. (2017). “A unified approach to interpreting model predictions,” in Advances in neural information processing systems (New York, NK: Curran Associates), 4765–4774.

[B29] Maxwell A. J. Ding J. You Y. Dong Z. Chehade H. Alvero A.. (2020). Identification of key signaling pathways induced by SARS-CoV2 that underlie thrombosis and vascular injury in COVID-19 patients. J. Leukoc. Biol. 10.1002/JLB.4COVR0920-552RR PMC7753679 33242368

[B30] Mira J. C. Gentile L. F. Mathias B. J. Efron P. A. Brakenridge S. C. Mohr A. M.. (2017). Sepsis Pathophysiology, Chronic Critical Illness, and Persistent Inflammation-Immunosuppression and Catabolism Syndrome. Crit. Care Med. 45, 253–262. 10.1097/CCM.0000000000002074 27632674 PMC5243156

[B31] Musher D. M. (2019). Pure viral sepsis secondary to community-acquired pneumonia in adults: risk and prognostic factors. J. Infect. Dis. 220, 1166–1171. 10.1093/infdis/jiz654 31115456 PMC7107497

[B32] Nat Nanotechnol (2020). Nanomedicine and the COVID-19 vaccines. Nat. Nanotechnol. 15 (12), 963 (2020). 10.1038/s41565-020-00820-0 33247210 PMC7692425

[B33] Ogunleye A. Wang Q.-G. (2020). XGBoost Model for Chronic Kidney Disease Diagnosis. IEEE/ACM Trans. Comput. Biol. Bioinf. 17, 2131–2140. 10.1109/TCBB.2019.2911071 30998478

[B34] Opoka-Winiarska V. Grywalska E. Rolinski J. (2020). Could hemophagocytic lymphohistiocytosis be the core issue of severe COVID-19 cases? BMC Med. 18, 214. 10.1186/s12916-020-01682-y 32664932 PMC7360379

[B35] Patel P. Walborn A. Rondina M. Fareed J. Hoppensteadt D. (2019). Markers of Inflammation and Infection in Sepsis and Disseminated Intravascular Coagulation. Clin. Appl. Thromb. Hemost. 25. 1076029619843338. 10.1177/1076029619843338 PMC6714897 30991817

[B36] Rosen D. A. Seki S. M. Fernandez-Castaneda A. Beiter R. M. Eccles J. D. Woodfolk J. A.. (2019). Modulation of the sigma-1 receptor-IRE1 pathway is beneficial in preclinical models of inflammation and sepsis. Sci. Transl. Med. 11, eaau5266. 10.1126/scitranslmed.aau5266 30728287 PMC6936250

[B37] Scheuplein V. A. Seifried J. Malczyk A. H. Miller L. Hocker L. Vergara-Alert J.. (2015). High secretion of interferons by human plasmacytoid dendritic cells upon recognition of Middle East respiratory syndrome coronavirus. J. Virol. 89, 3859–3869. 10.1128/JVI.03607-14 25609809 PMC4403407

[B38] Shen J. Liu L. Zhang F. Gu J. Pan G. (2019). LncRNA TapSAKI promotes inflammation injury in HK-2 cells and urine derived sepsis-induced kidney injury. J. Pharm. Pharmacol. 71, 839–848. 10.1111/jphp.13049 30666657

[B39] Singer M. Deutschman C. S. Seymour C. W. Shankar-Hari M. Annane D. Bauer M.. (2016). The Third International Consensus Definitions for Sepsis and Septic Shock (Sepsis-3). JAMA 315, 801–810. 10.1001/jama.2016.0287 26903338 PMC4968574

[B40] Tang N. Li D. Wang X. Sun Z. (2020). Abnormal coagulation parameters are associated with poor prognosis in patients with novel coronavirus pneumonia. J. Thromb. Haemost. 18, 844–847. 10.1111/jth.14768 32073213 PMC7166509

[B41] Taylor R. A. Pare J. R. Venkatesh A. K. Mowafi H. Melnick E. R. Fleischman W.. (2016). Prediction of In-hospital Mortality in Emergency Department Patients With Sepsis: A Local Big Data–Driven, Machine Learning Approach. Acad. Emergency Med. 23, 269–278. 10.1111/acem.12876 PMC5884101 26679719

[B42] The Lancet (2020). COVID-19 vaccines: no time for complacency. Lancet 396, 1607. 10.1016/S0140-6736(20)32472-7 33220729 PMC7834228

[B43] Tonekaboni S. Joshi S. McCradden M. D. Goldenberg A. (2019). What clinicians want: contextualizing explainable machine learning for clinical end use. Proc. Machine Learning Res. 1–21.

[B44] Tu Y. F. Chien C. S. Yarmishyn A. A. Lin Y. Y. Luo Y. H. Lin Y. T.. (2020). A Review of SARS-CoV-2 and the Ongoing Clinical Trials. Int. J. Mol. Sci. 21, 2657. 10.3390/ijms21072657 PMC7177898 32290293

[B45] Tynell J. Westenius V. Ronkko E. Munster V. J. Melen K. Osterlund P.. (2016). Middle East respiratory syndrome coronavirus shows poor replication but significant induction of antiviral responses in human monocyte-derived macrophages and dendritic cells. J. Gen. Virol. 97, 344–355. 10.1099/jgv.0.000351 26602089 PMC4804640

[B46] Vlachodimitropoulou Koumoutsea E. Vivanti A. J. Shehata N. Benachi A. Le Gouez A. Desconclois C.. (2020). COVID19 and acute coagulopathy in pregnancy. J. Thromb. Haemost. 18, 1648–1652. 10.1111/jth.14856 32302459 PMC9770955

[B47] Yao R.-q. Jin X. Wang G.-w. Yu Y. Wu G.-s. Zhu Y.-b.. (2020). A Machine Learning-Based Prediction of Hospital Mortality in Patients With Postoperative Sepsis. Front. Med. 7, 445. 10.3389/fmed.2020.00445 PMC7438711 32903618

[B48] Ye Q. Wang B. Mao J. (2020). The pathogenesis and treatment of the ‘Cytokine Storm’ in COVID-19. J. Infect. 80, 607–613. 10.1016/j.jinf.2020.03.037 32283152 PMC7194613

[B49] Zabihi M. Kiranyaz S. Gabbouj M. (2019). Sepsis Prediction in Intensive Care Unit Using Ensemble of XGboost Models. in Computing in Cardiology (CinC) (Singapore), 1–4. 10.22489/CinC.2019.238

[B50] Zhang C. Shi L. Wang F. S. (2020). Liver injury in COVID-19: management and challenges. Lancet Gastroenterol. Hepatol. 5, 428–430. 10.1016/S2468-1253(20)30057-1 32145190 PMC7129165

[B51] Zhou W. Liu Y. Tian D. Wang C. Wang S. Cheng J.. (2020). Potential benefits of precise corticosteroids therapy for severe 2019-nCoV pneumonia. Signal Transduct. Target. Ther. 5, 18. 10.1038/s41392-020-0127-9 32296012 PMC7035340

