# Supplementary figures and images for: Prediction of Sepsis in COVID-19 Using Laboratory Indicators
Source: Front Cell Infect Microbiol. 2021 Mar 2;10:586054. doi: 10.3389/fcimb.2020.586054 (PMC7966961; doi:10.3389/fcimb.2020.586054)

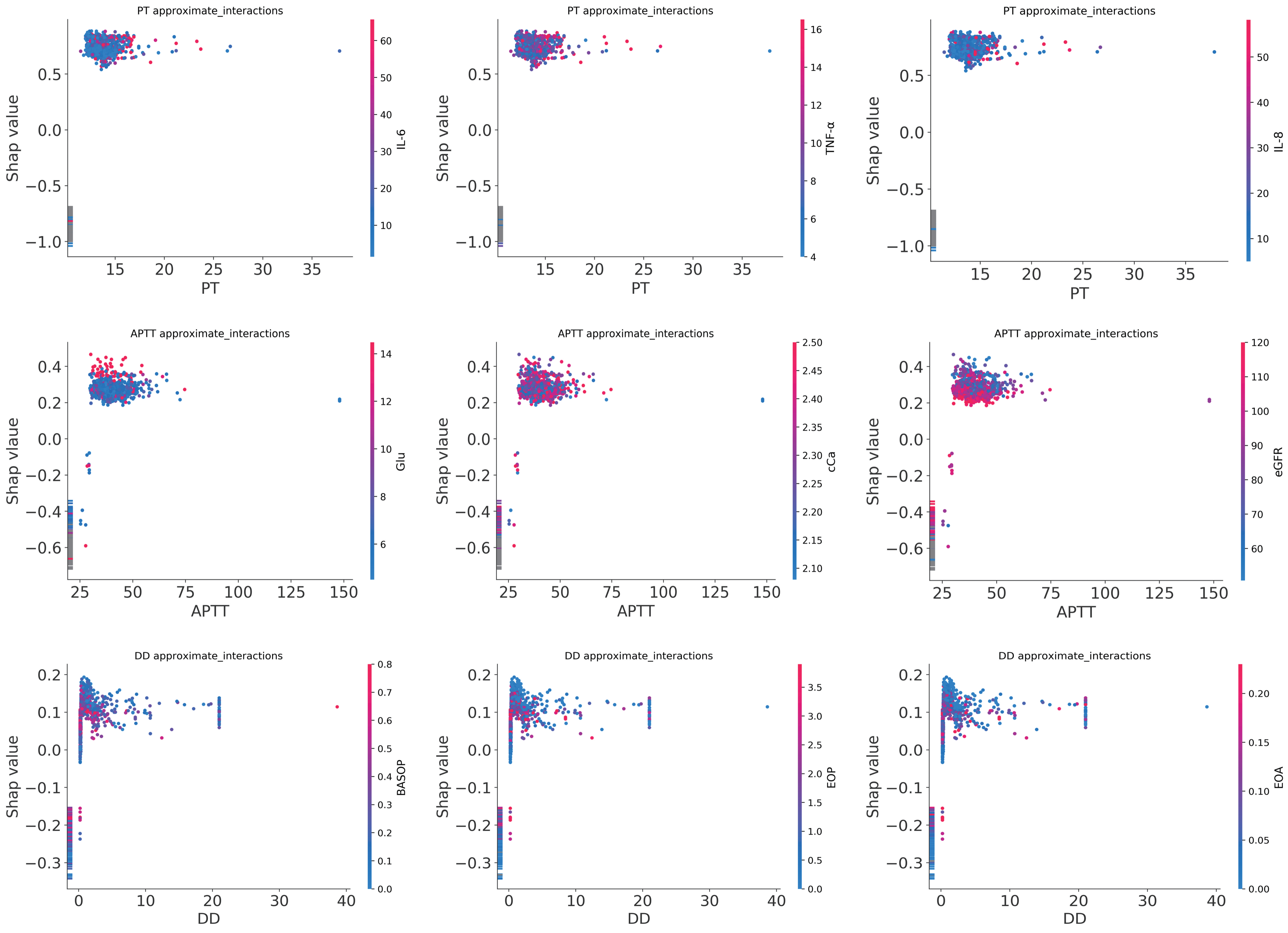

Supplement: Supplementary file 1 [file DataSheet_1.pdf]

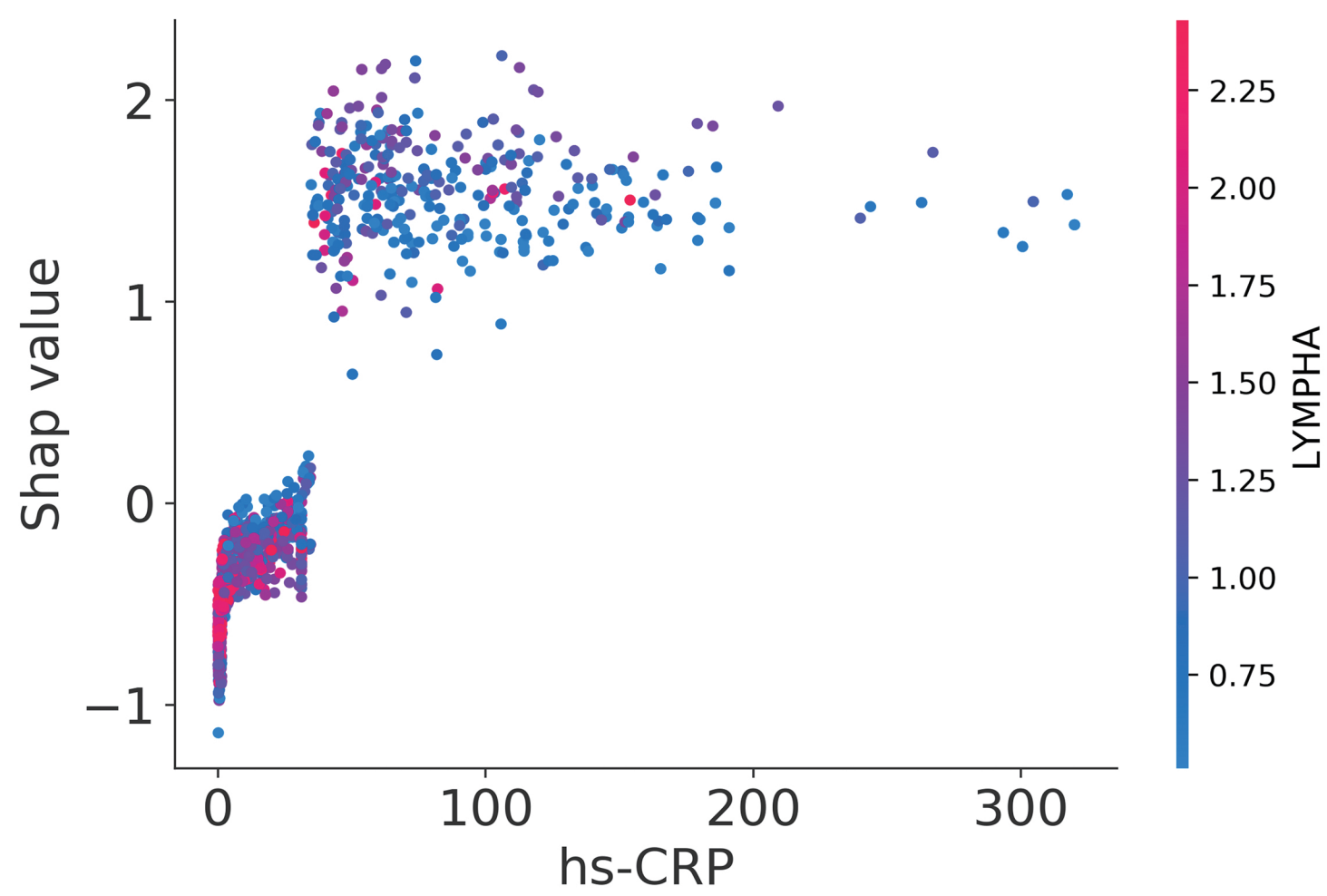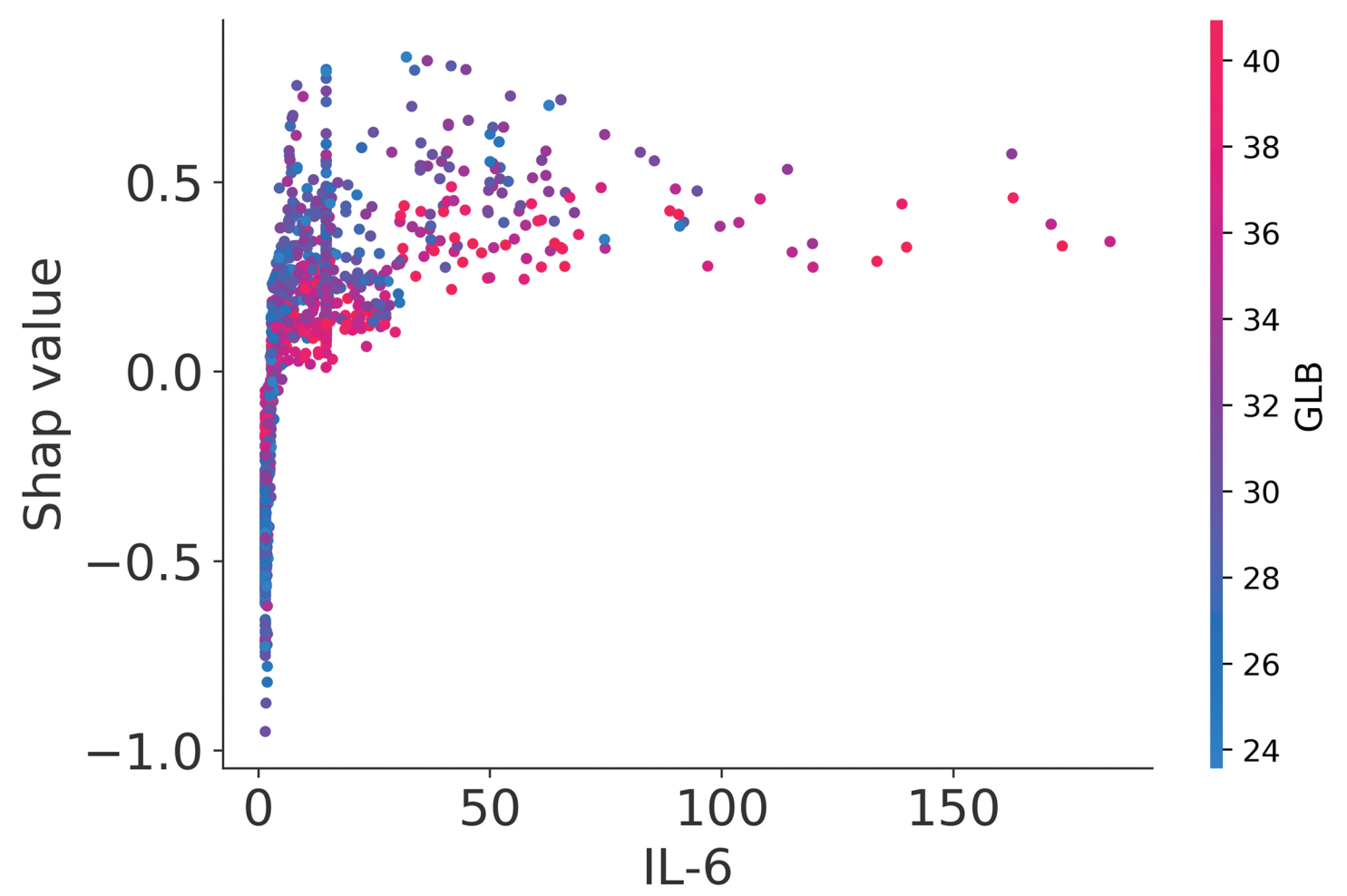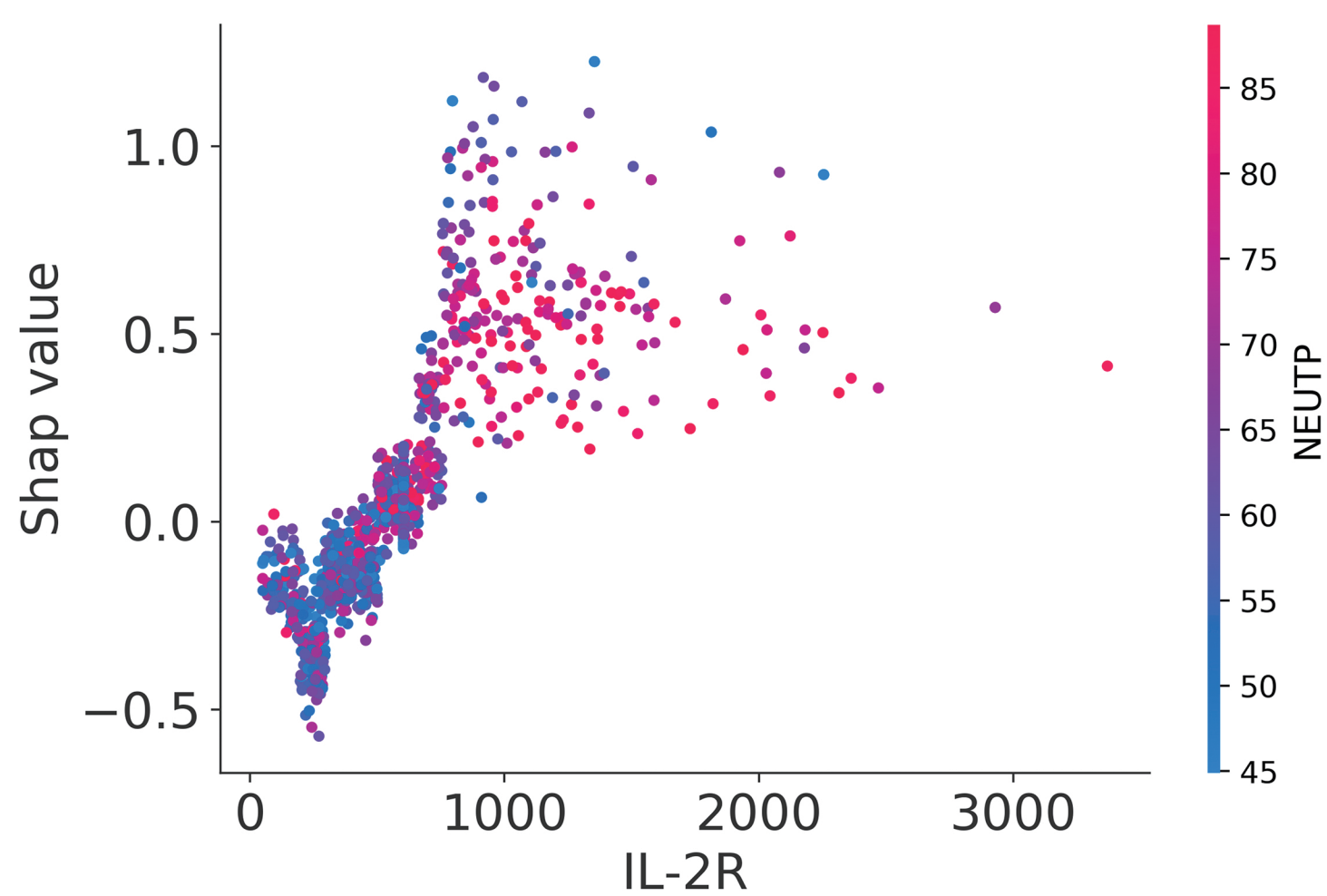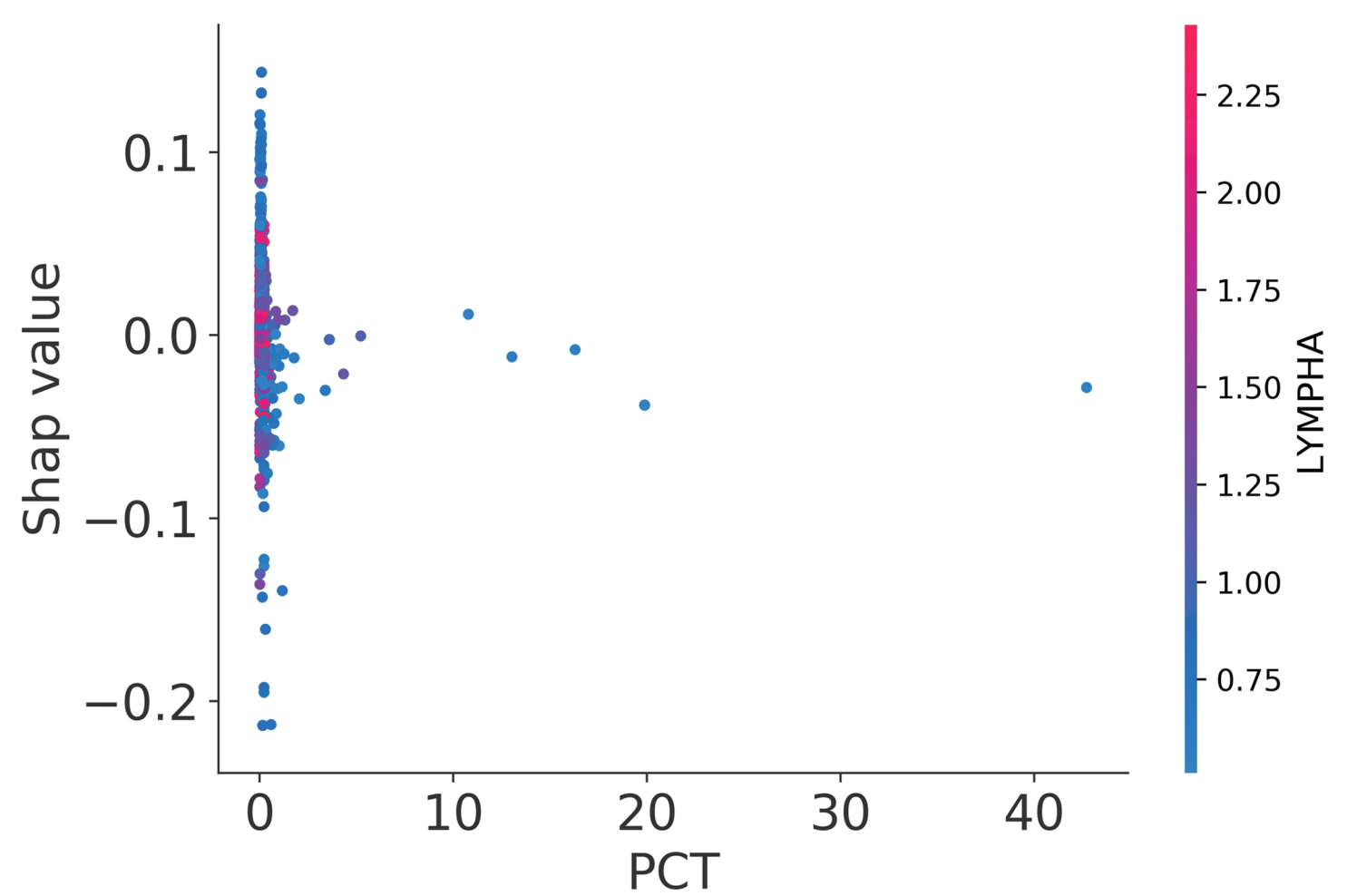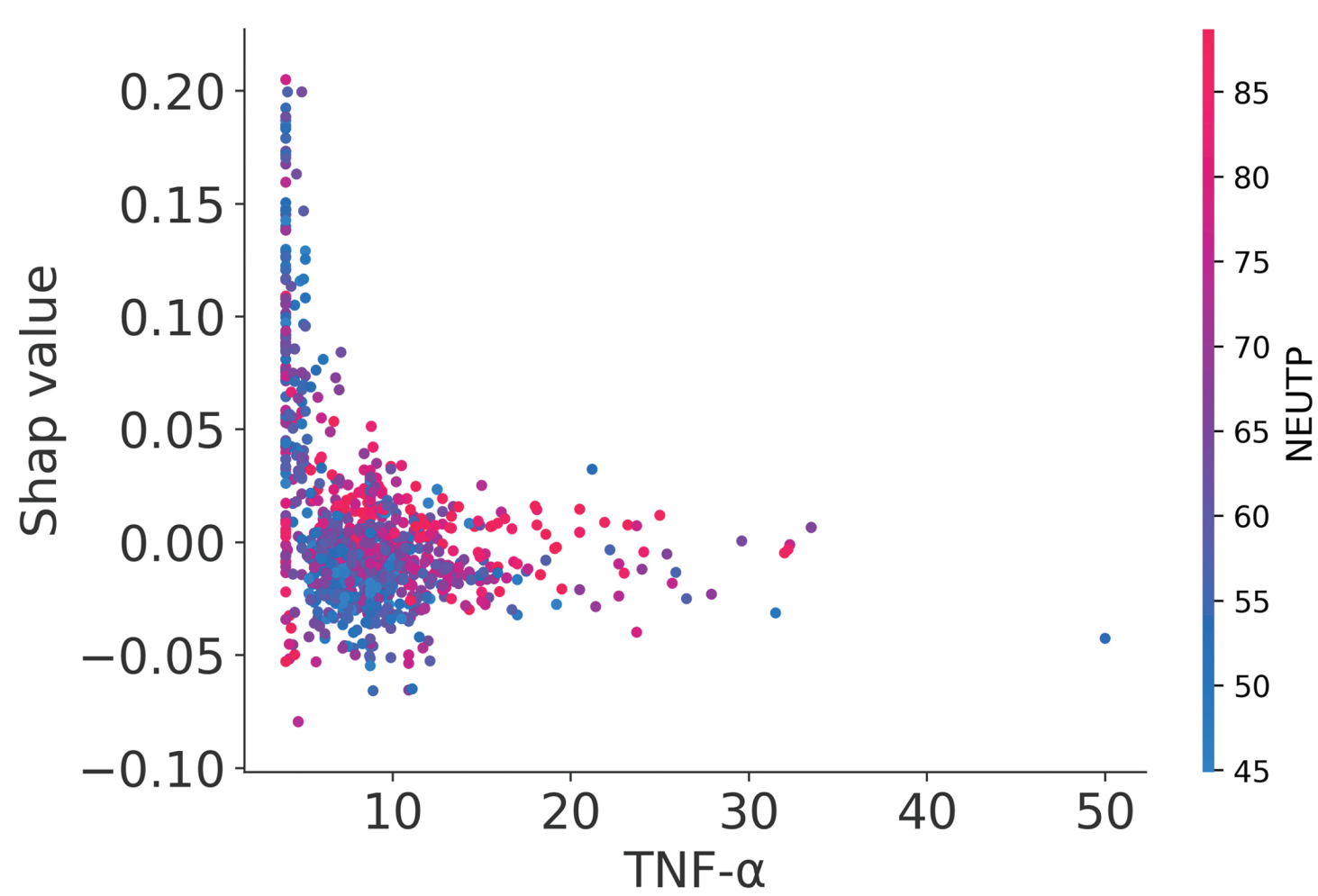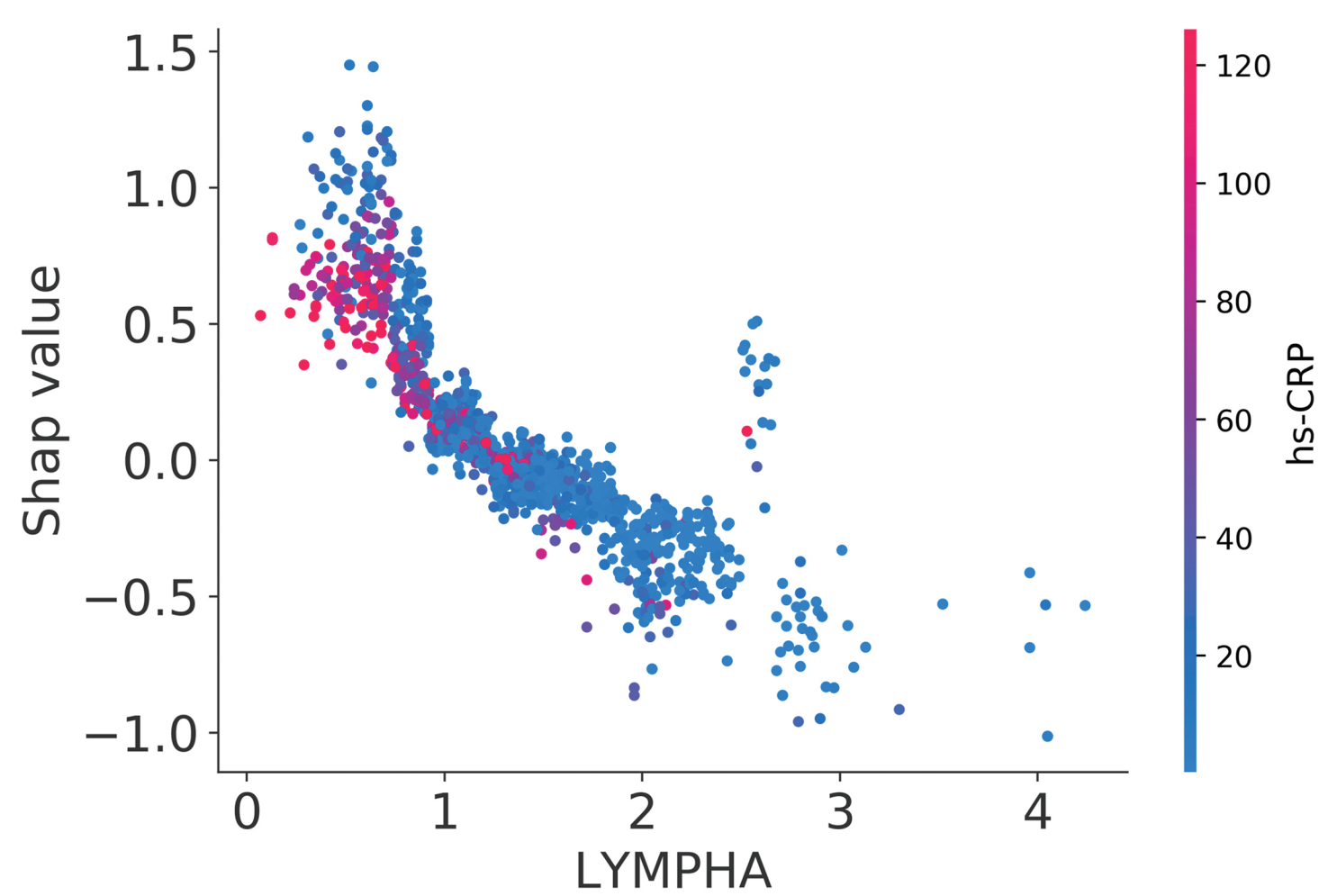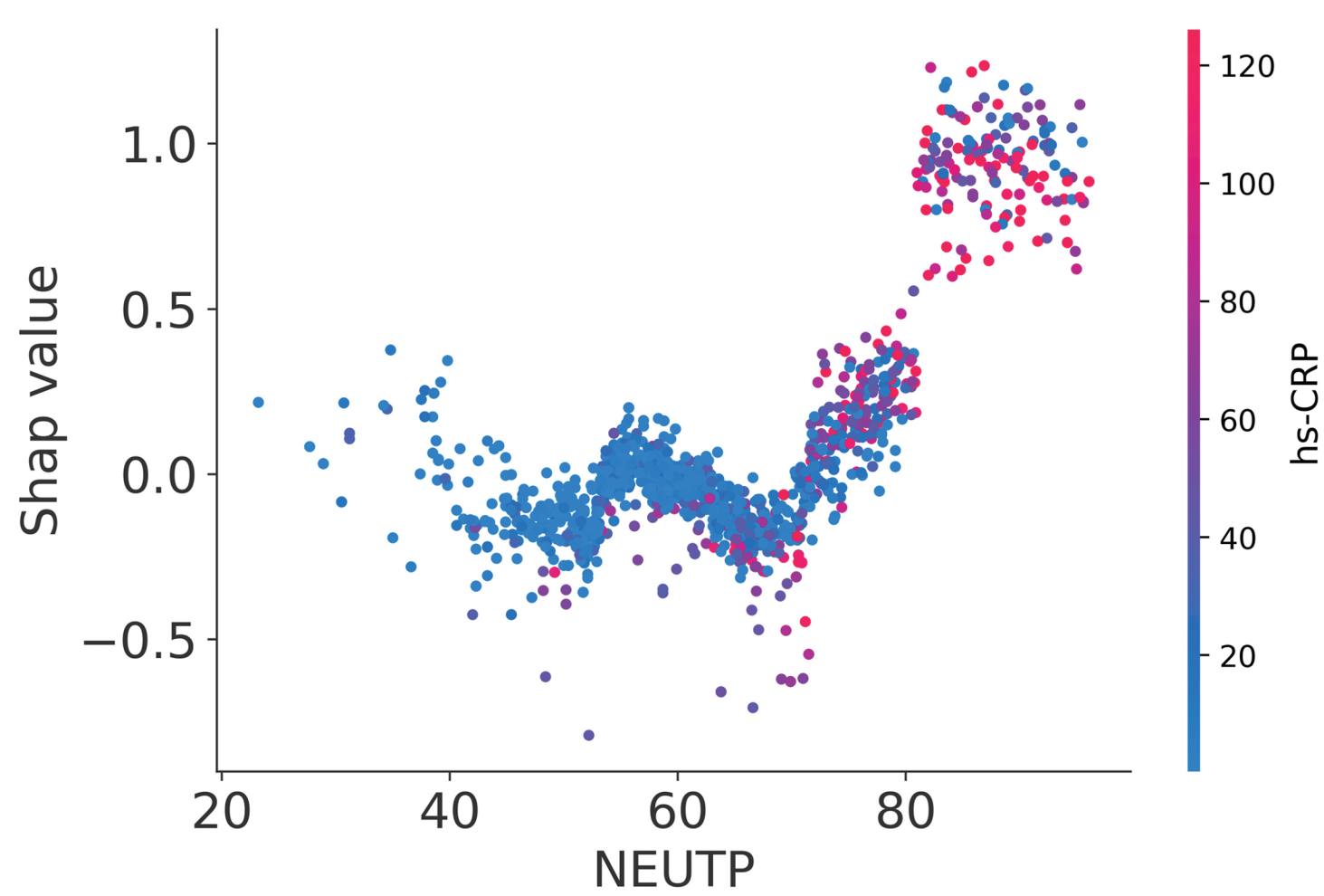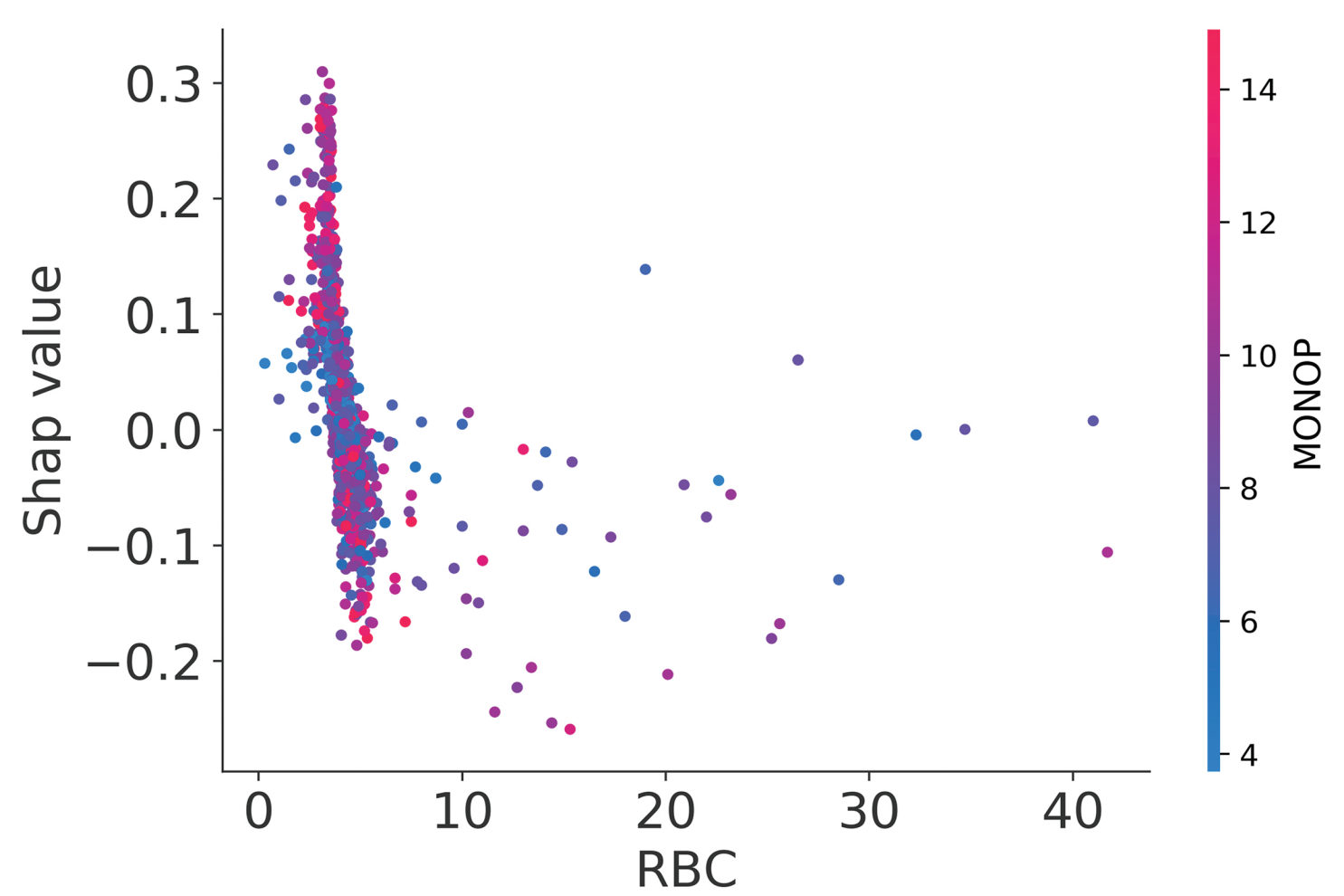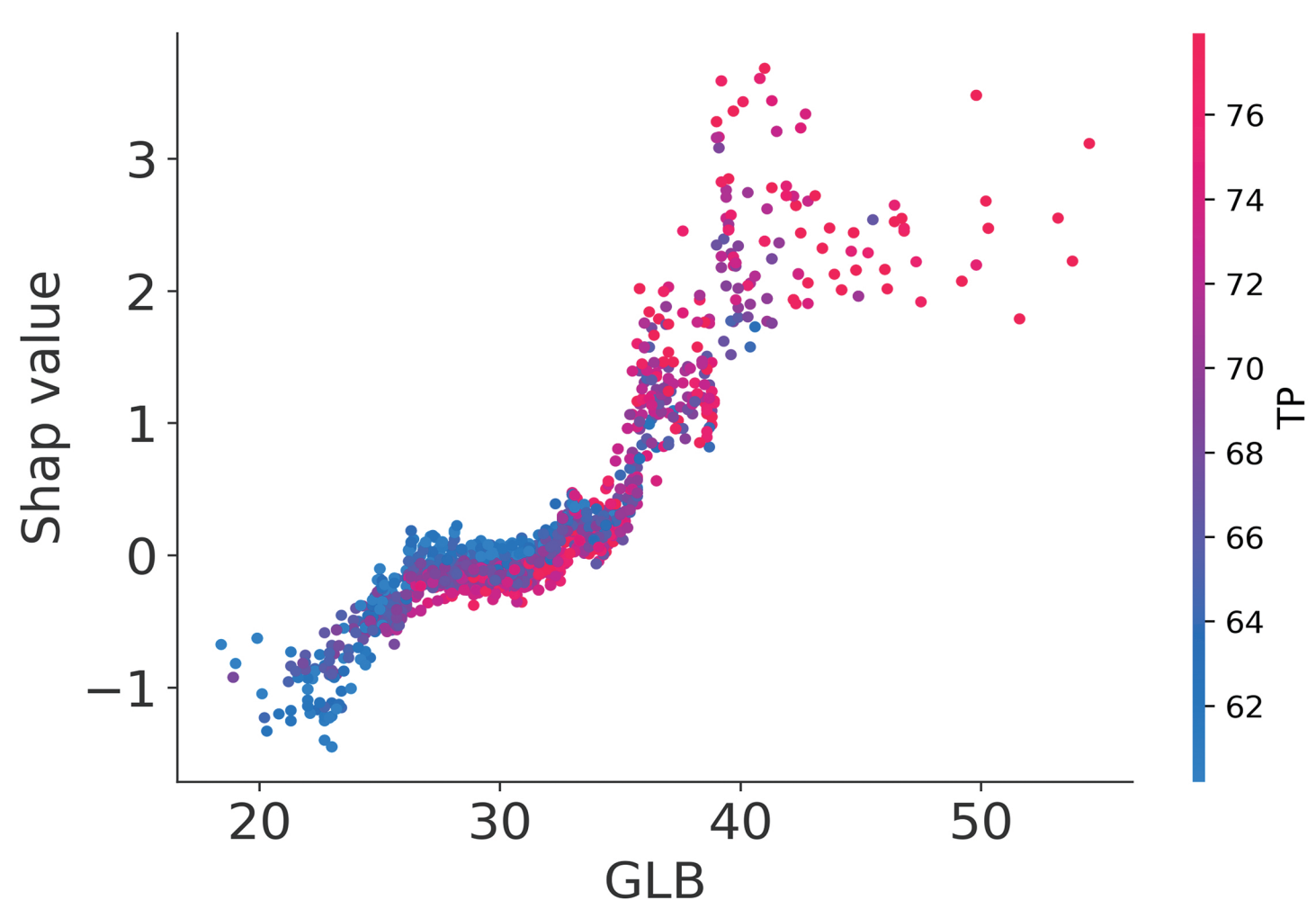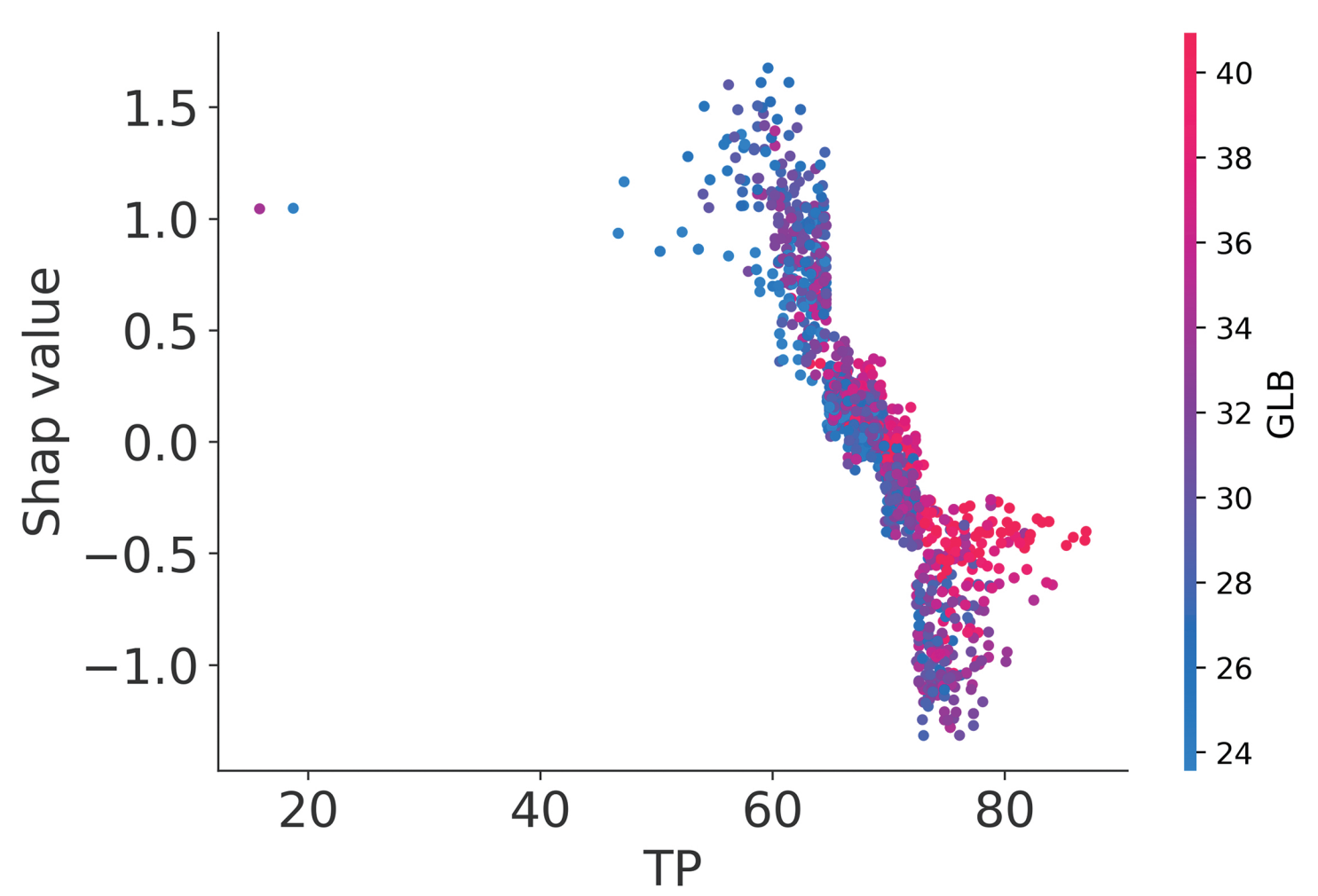

Supplement: Supplementary file 2 [file DataSheet_2.pdf]

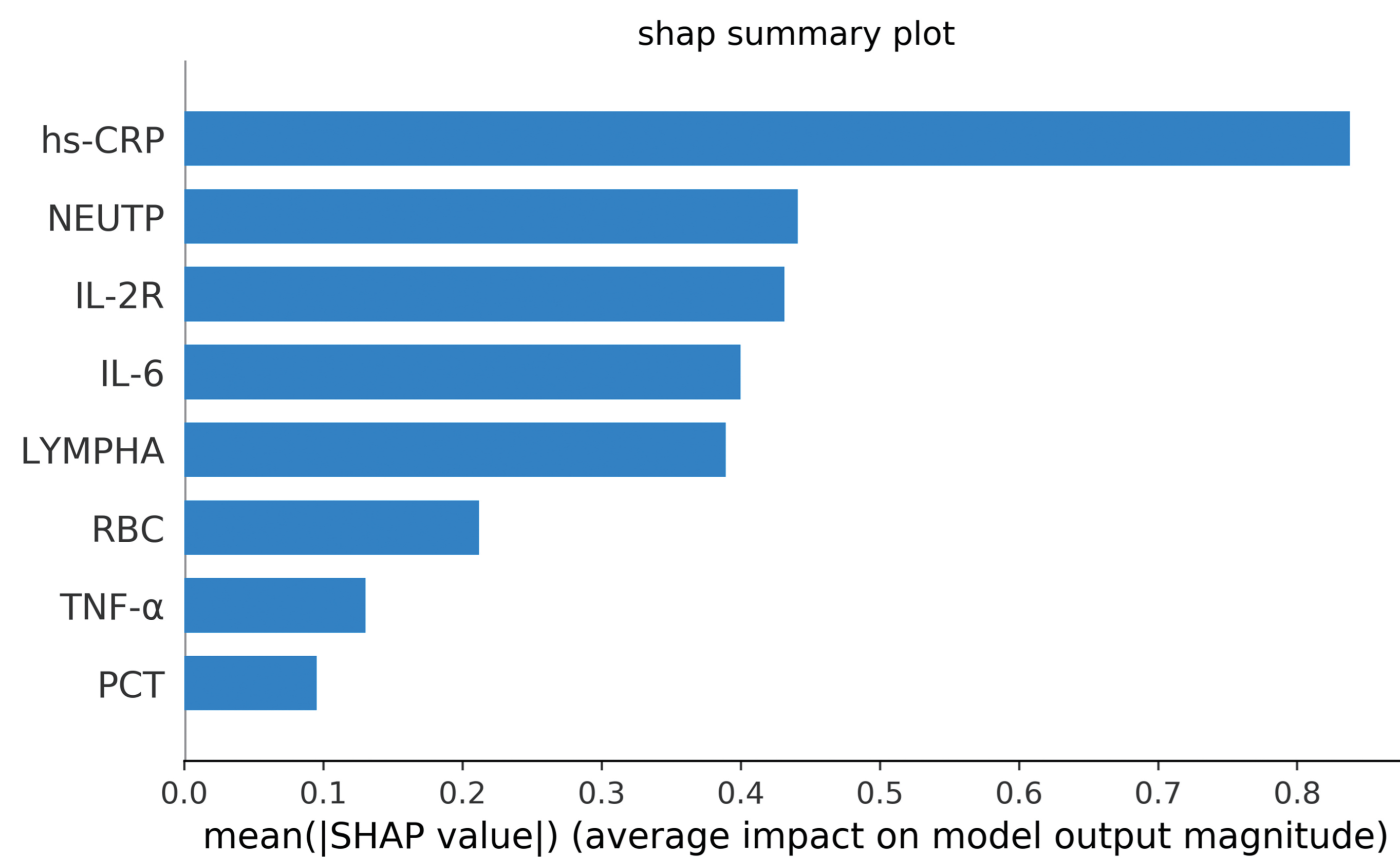

(a)

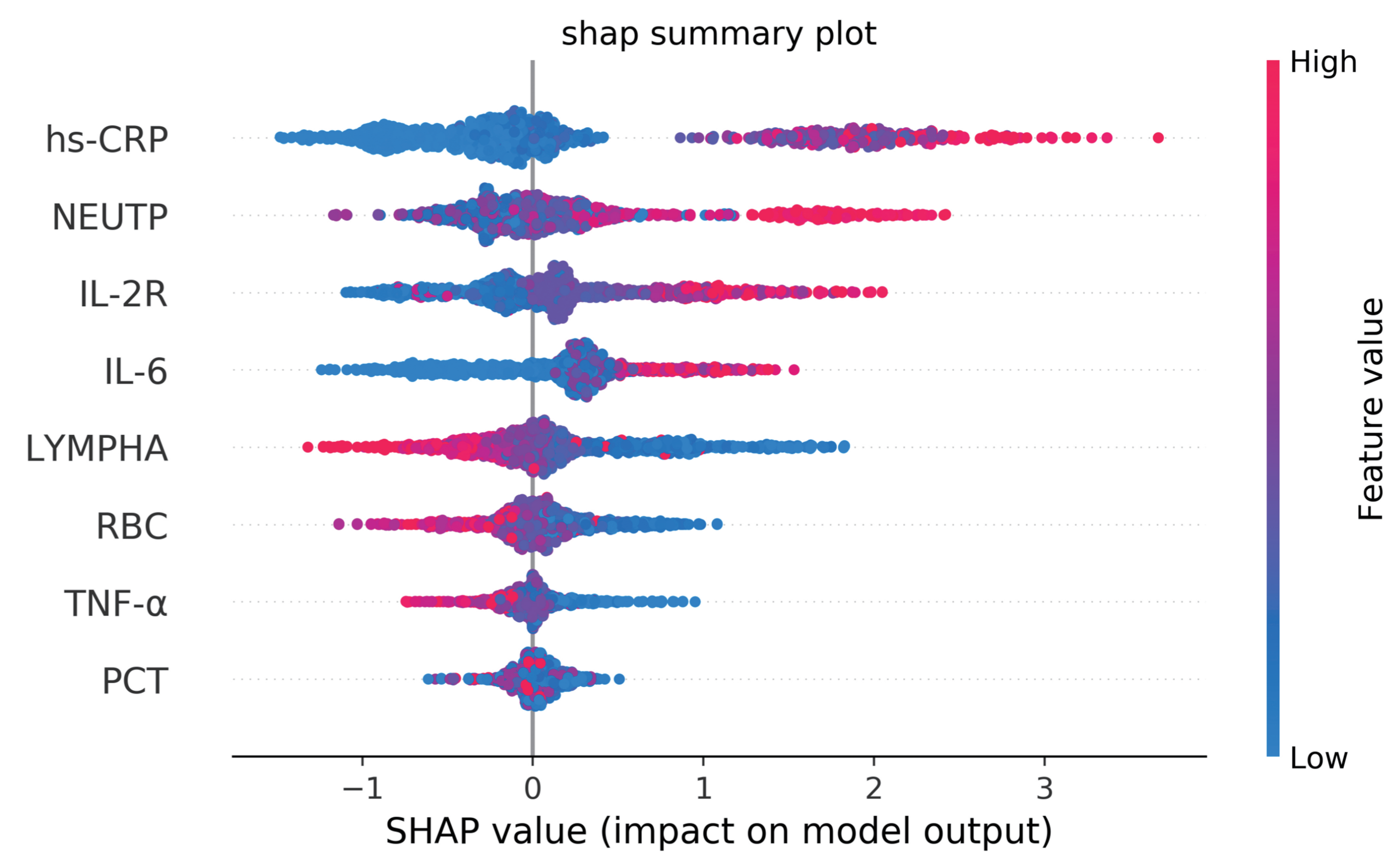

(b)

Supplement: Supplementary file 3 [file DataSheet_3.pdf]

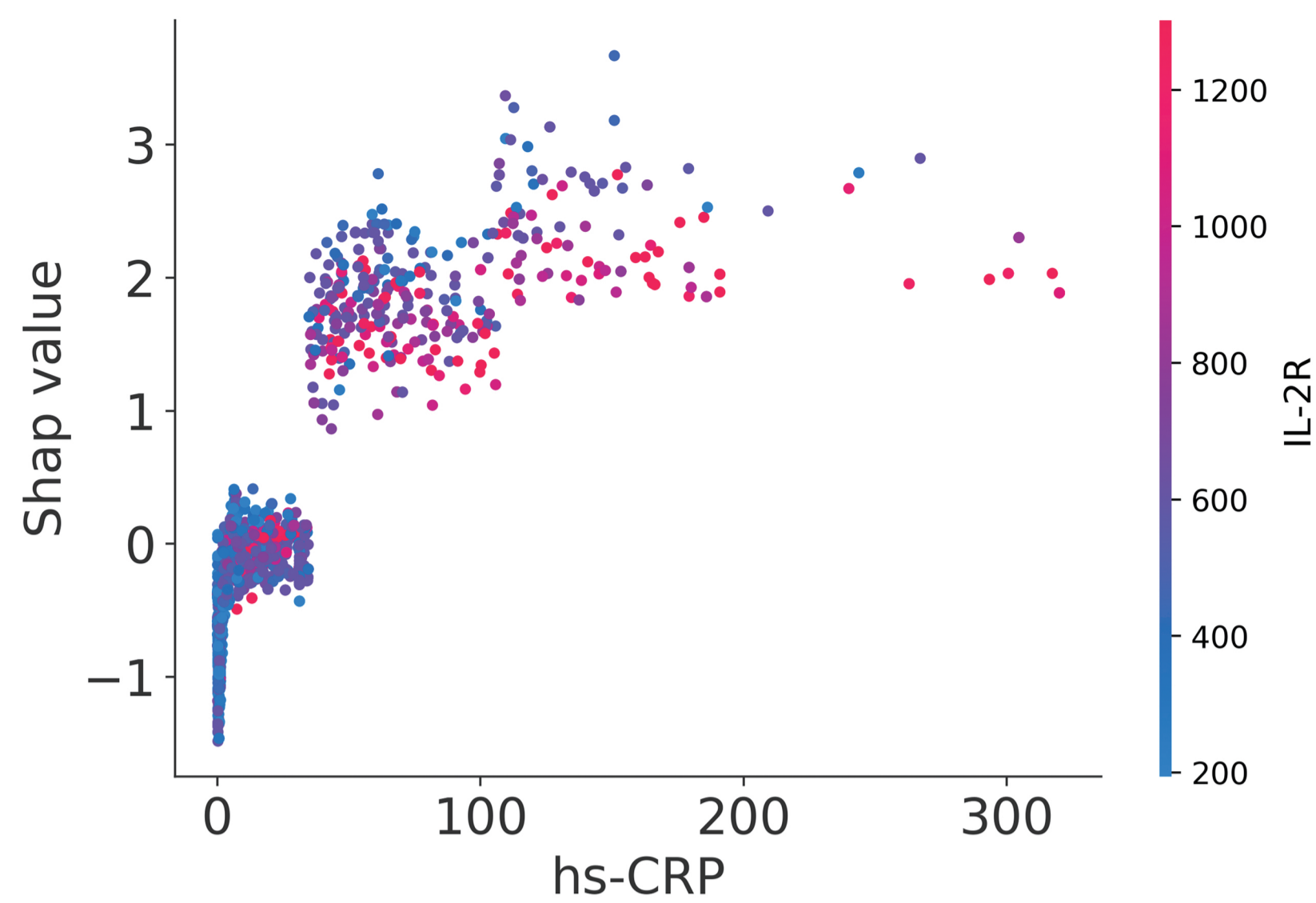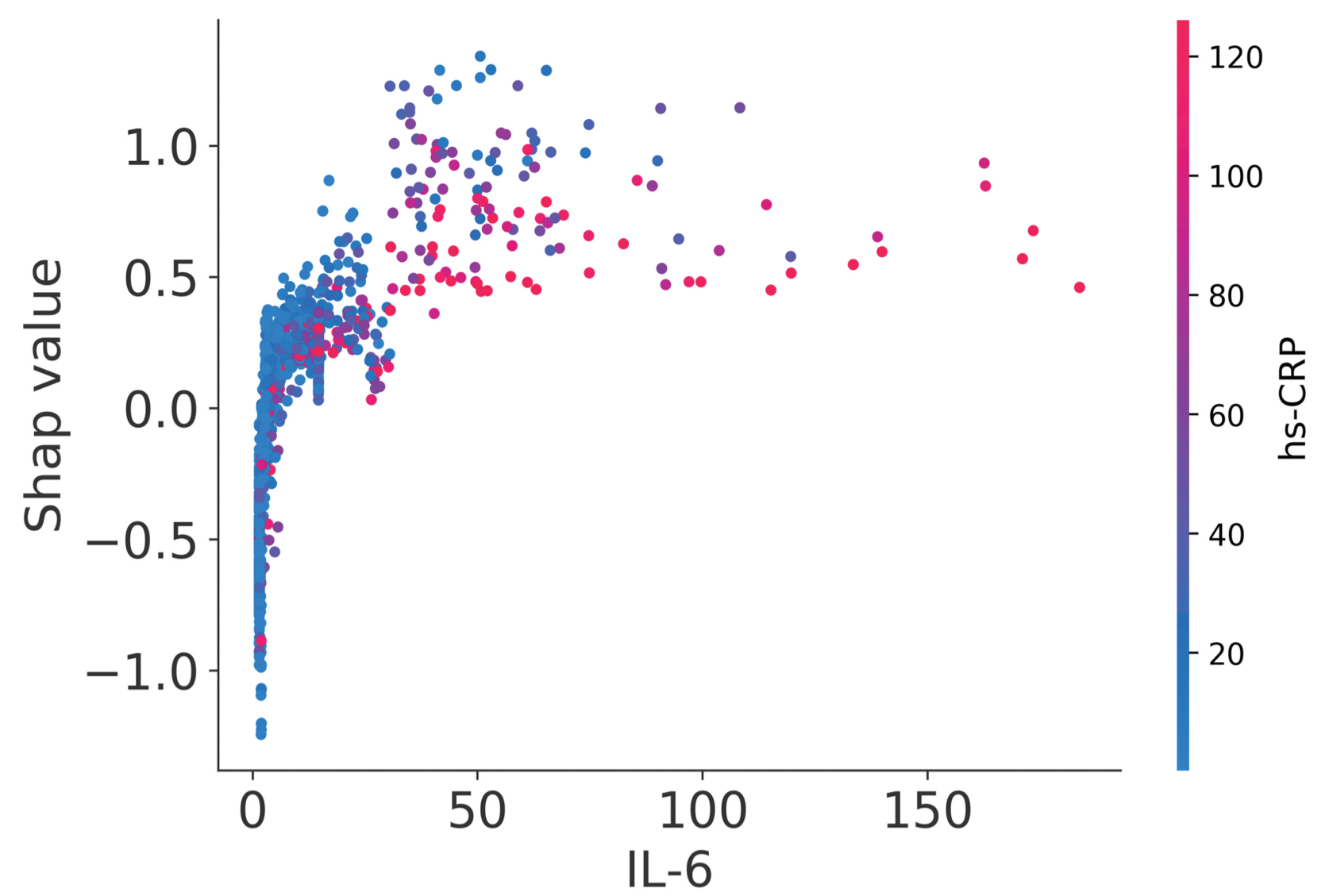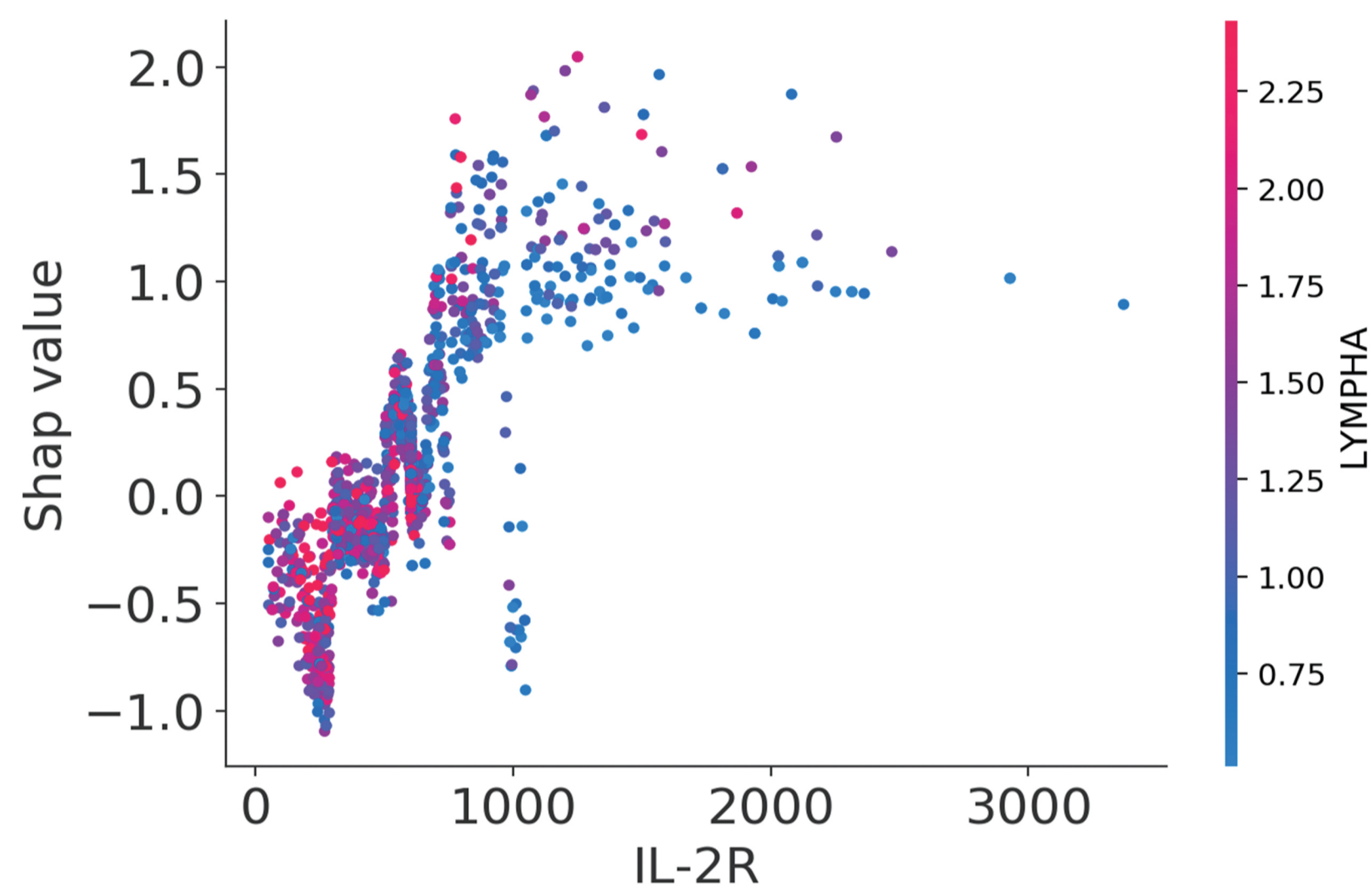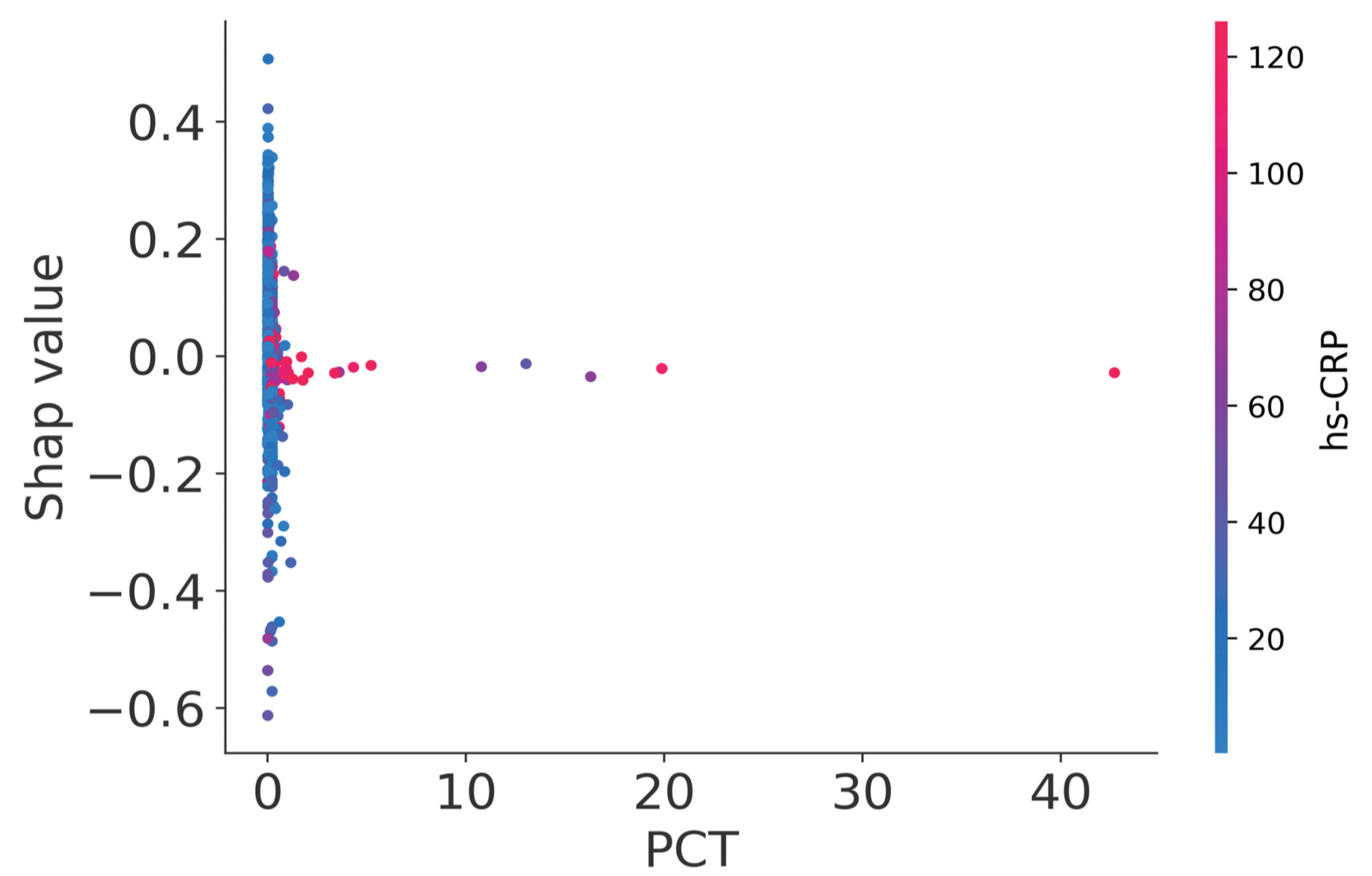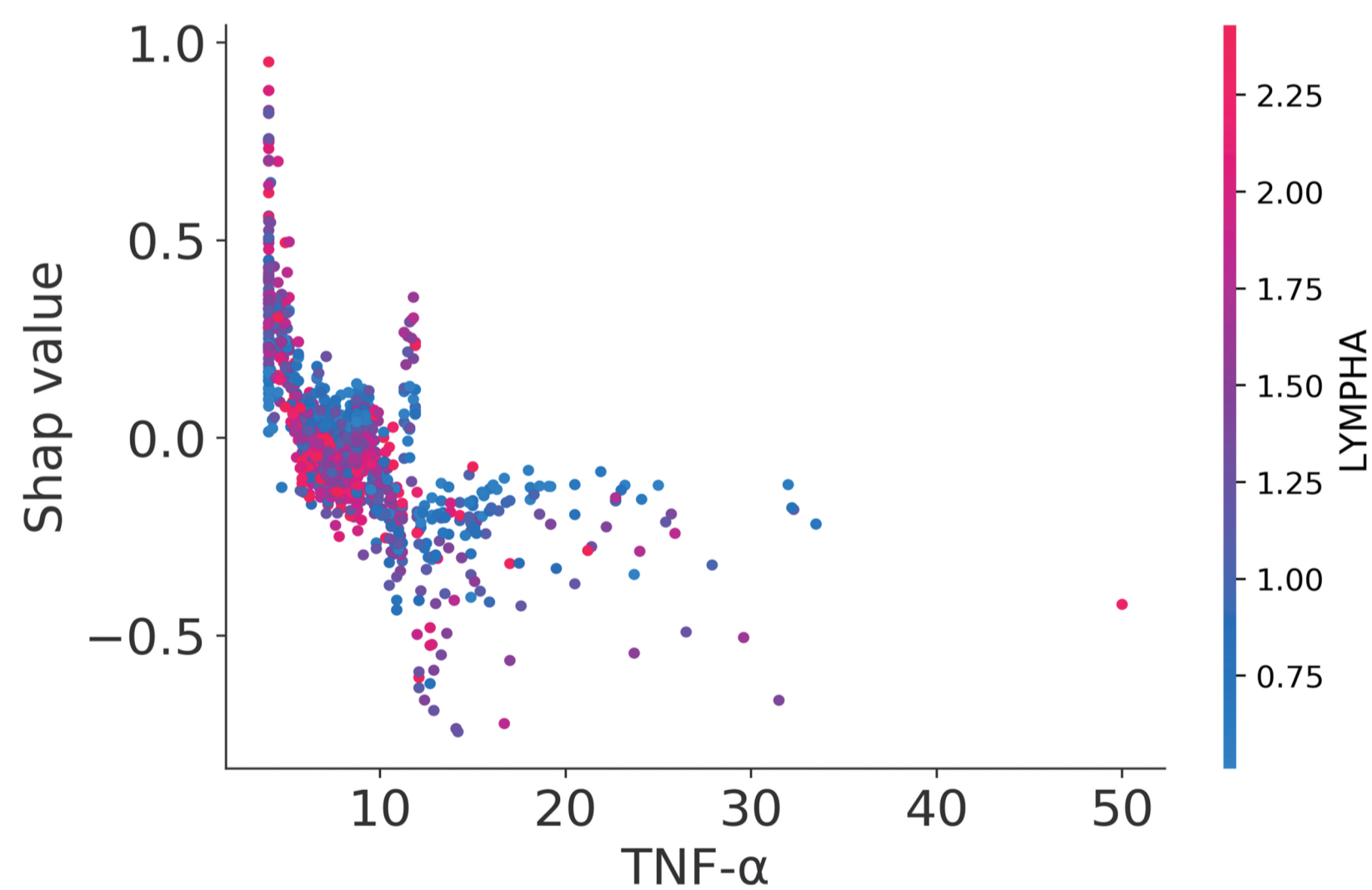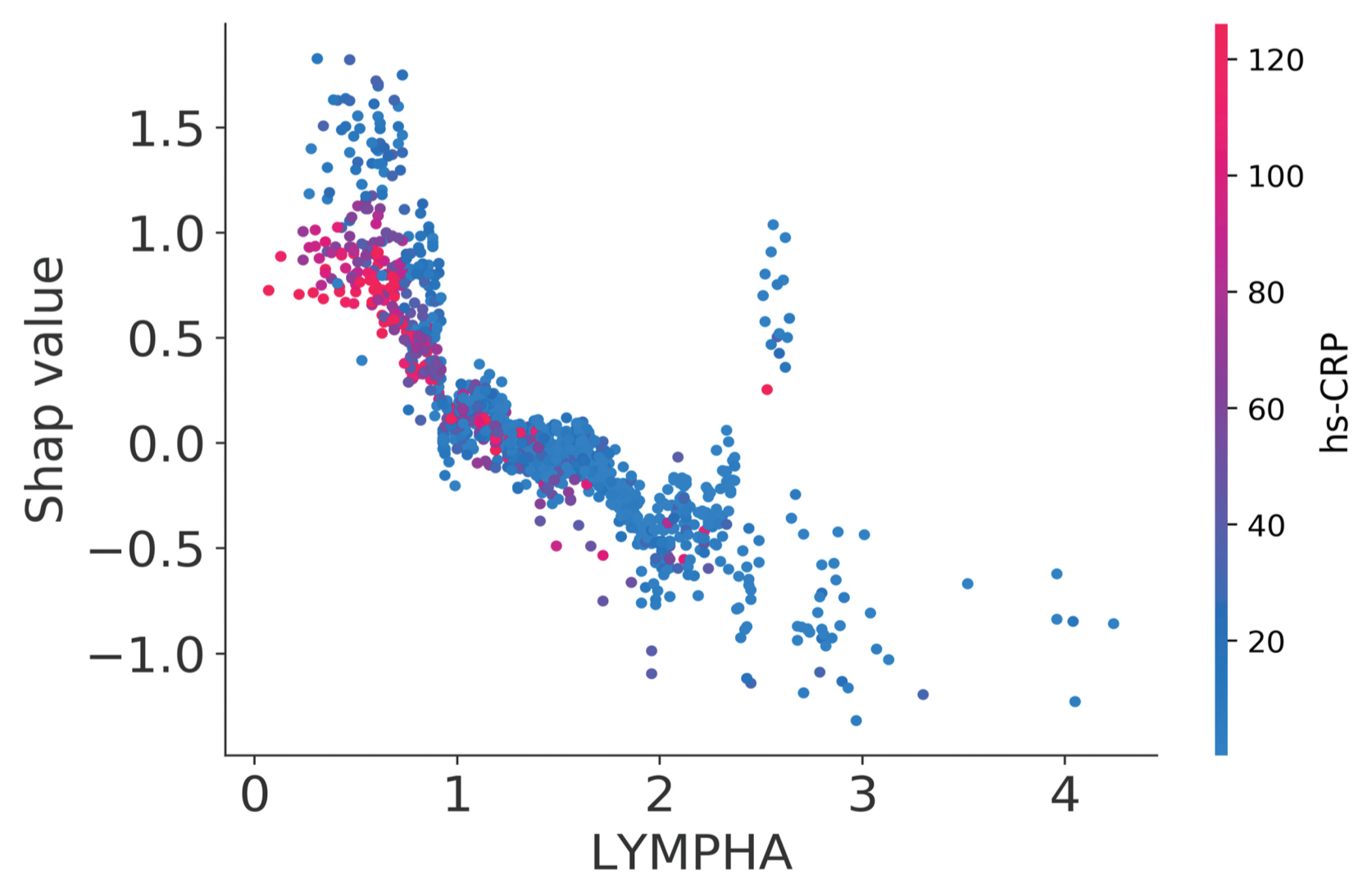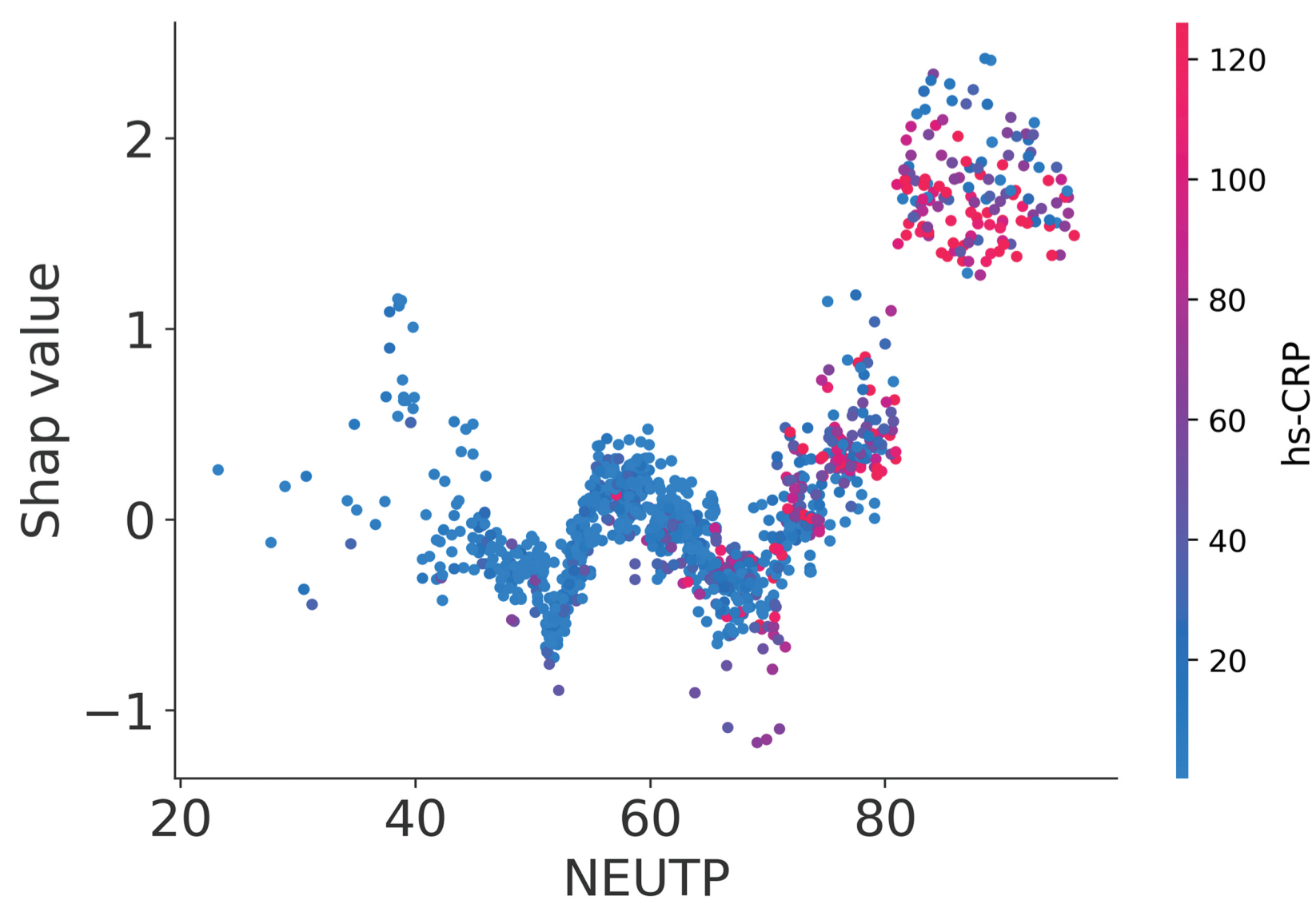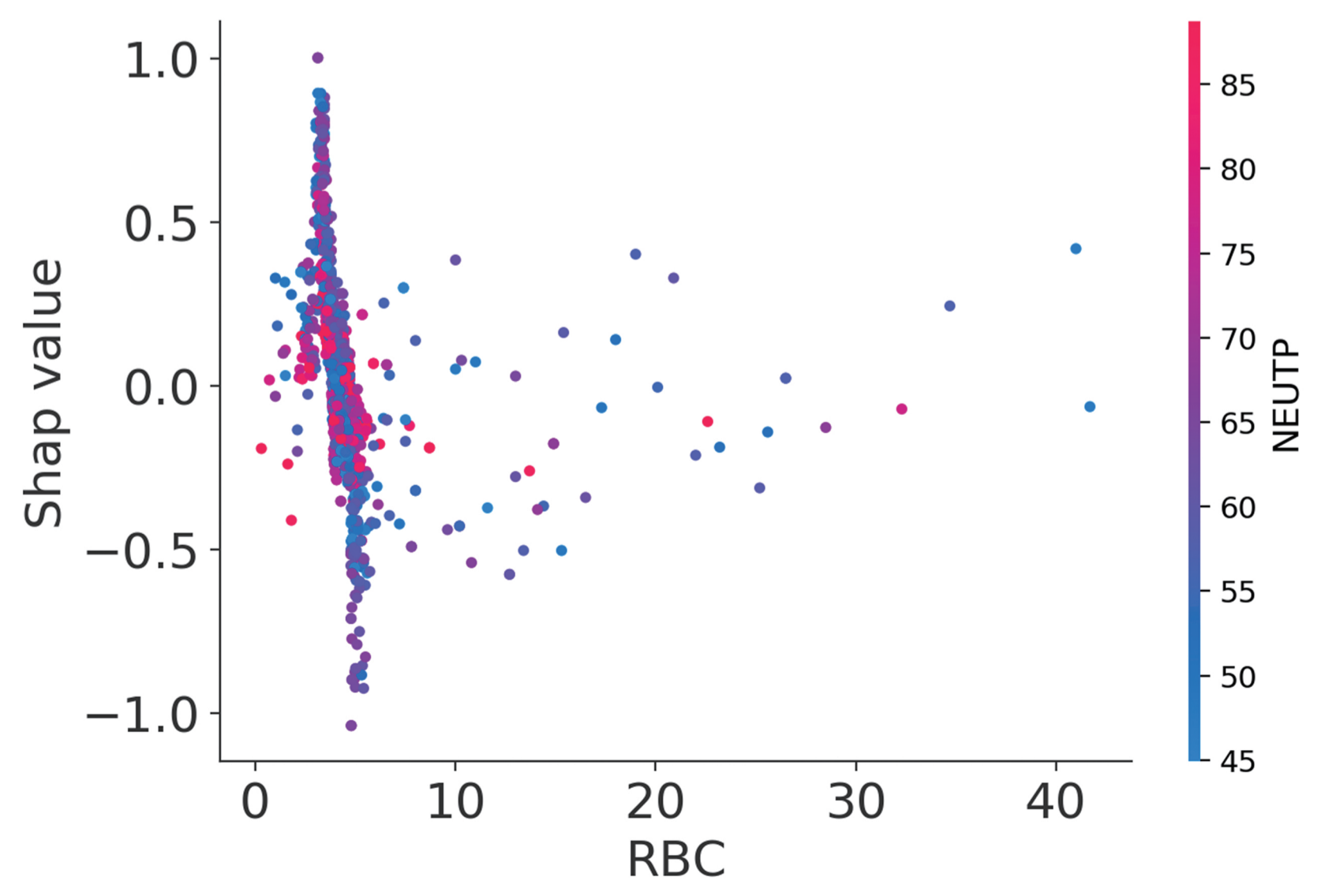

Supplement: Supplementary file 4 [file DataSheet_4.pdf]
