# Supplementary material for: Prediction of Sepsis in COVID-19 Using Laboratory Indicators
Source: Front Cell Infect Microbiol. 2021 Mar 2;10:586054. doi: 10.3389/fcimb.2020.586054 (PMC7966961; doi:10.3389/fcimb.2020.586054)
Supplement: Supplementary file 5 [file Table_1.docx]

| Index | Type | Name |
| --- | --- | --- |
| 1 | Inflammatory indicators | hypersensitive C-reactive protein (hs-CRP), procalcitonin (PCT), Interleukin 6 (IL-6), Interleukin 1 (IL-1), tumor necrosis factor-α (TNF-α), Interleukin2 Receptor (IL-2 receptor), Interleukin 8 (IL-8), Interleukin 10 (IL-10) |
| 2 | Coagulation function indicators | Prothrombin time (PT), Prothrombin activity (PTA),  Activated partial thromboplastin time (APTT), Thrombin time (Tt),  International standardized ratio (INR), D-dimer (DD), Fibrinogen (FIB) |
| 3 | Blood routine indicators | Neutrophil percentage (NEUTP), Neutrophil absolute value (NEUTA), Lymphocyte percentage (LYMPHP), Lymphocyte absolute value (LYMPHA), Monocytes percentage (MONOP), Monocytes absolute value (MONOA), Eosinophilia percentage (EOP), Eosinophilia absolute value (EOA), Basophil percentage (BASOP), Basophil absolute value (BASOA), Red blood cell count (RBC), Hematocrit (HCT), Mean red blood cell volume (MCV), Mean corpuscular hemoglobinv (MCH), Mean corpuscular hemoglobin concentration (MCHC), Red Blood Cell Distribution Width CV (RDWC), Red Blood Cell Distribution SD (RDWS), Platelet count (PLT), Platelet distribution width (PDW), Mean platelet volume (MPV), Platelet-large cell ratio (PLCR), Plateletcrit (THR), hemoglobin (HB) |
| 4 | Biochemistry indicators | Amylase (AMYL), Magnesium (Mg), phosphorus (Phos), Triglyceride (TG), globulin (GLB), calibration Calcium (cCa), Alkaline phosphatase (ALPL), actate dehydrogenase (LDH)，Alanine transaminase（ALT），sodium（Na），Prealbumin (PAB), Total bile acids (TBA), Total bilirubin (TBil), cholestrerol (Chol), Total protein (TP), Aspartate aminotransferase (AST), Calcium (Ga), 5-nucleotidase(dNTP), y-Glutamyl transferase (GLUta), HCO_3_(HCO), Cholinseterase (Che), chlorine (chlor), Directbilirubin (Dbil), albumin (ALB), potassium (pota), Creatinine (Cr), Urea (Urea), Uric acid (UA), estimated Glomerular filtration rate (eGFR), Glucose (Glu), Creatine Kinase (CK) |

***Supplementary Table* 1:** **Risk factors used in the analysis of VSCS-2**. The 69 features were used in the first calssification model, and the 45 features (underlined) were features used in the first preditive model.

## Supplementary Figures

**Figure 1**: **Single risk factors analysis by SHAP dependence plot in the first classification model.** For the three coagulation indexes with the highest influence, PT, APTT, and DD, three single risk factors analysis charts, including correlation analysis (sub-ordinate), are presented respectively. For example, the three laboratory indexes with the highest correlation with PT are IL-6, IL-6, TNF-, and IL-8. The abscisic coordinate in each figure is the feature's SHAP value, and the ordinate is the SHAP value of the sample under the value of the feature. When SHAP value is greater than 0, it indicates that this feature improves the predicted value and has a positive effect. On the contrary, it indicates that this feature reduces the predicted value and has an adverse effect.

**Figure 2**: **The single risk factors analysis by SHAP dependence plot in the first predictive model.**

**Figure 3**: **The features importance were obtained by SHAP analysis in the second predictive modle.** (a) The feature's importance of the first predictive model based on additivity. (b) The overall analysis of the influence of each feature.

**Figure 4**: **The single risk factor analysis by SHAP dependence plot in the second predictive model.**
